# Recommendations for the Use of Automated Gray Matter Segmentation Tools: Evidence from Huntington’s Disease

**DOI:** 10.3389/fneur.2017.00519

**Published:** 2017-10-10

**Authors:** Eileanoir B. Johnson, Sarah Gregory, Hans J. Johnson, Alexandra Durr, Blair R. Leavitt, Raymund A. Roos, Geraint Rees, Sarah J. Tabrizi, Rachael I. Scahill

**Affiliations:** ^1^Huntington’s Disease Centre, UCL Institute of Neurology, University College London, London, United Kingdom; ^2^Department of Electrical and Computer Engineering, University of Iowa, Iowa City, IA, United States; ^3^Department of Genetics and Cytogenetics, INSERMUMR S679, APHP, ICM Institute, Hôpital de la Salpêtrière, Paris, France; ^4^Centre for Molecular Medicine and Therapeutics, Department of Medical Genetics, University of British Columbia, Vancouver, BC, Canada; ^5^Department of Neurology, Leiden University Medical Centre, Leiden, Netherlands; ^6^George-Huntington-Institut, münster, Germany; ^7^Wellcome Trust Centre for Neuroimaging, University College London, London, United Kingdom

**Keywords:** gray matter, segmentation, Huntington’s disease, FreeSurfer, statistical parametric mapping, advanced normalization tools, FMRIB’s Software Library, Multi-Atlas Label Propagation with Expectation–Maximization-based refinement

## Abstract

The selection of an appropriate segmentation tool is a challenge facing any researcher aiming to measure gray matter (GM) volume. Many tools have been compared, yet there is currently no method that can be recommended above all others; in particular, there is a lack of validation in disease cohorts. This work utilizes a clinical dataset to conduct an extensive comparison of segmentation tools. Our results confirm that all tools have advantages and disadvantages, and we present a series of considerations that may be of use when selecting a GM segmentation method, rather than a ranking of these tools. Seven segmentation tools were compared using 3 T MRI data from 20 controls, 40 premanifest Huntington’s disease (HD), and 40 early HD participants. Segmented volumes underwent detailed visual quality control. Reliability and repeatability of total, cortical, and lobular GM were investigated in repeated baseline scans. The relationship between each tool was also examined. Longitudinal within-group change over 3 years was assessed *via* generalized least squares regression to determine sensitivity of each tool to disease effects. Visual quality control and raw volumes highlighted large variability between tools, especially in occipital and temporal regions. Most tools showed reliable performance and the volumes were generally correlated. Results for longitudinal within-group change varied between tools, especially within lobular regions. These differences highlight the need for careful selection of segmentation methods in clinical neuroimaging studies. This guide acts as a primer aimed at the novice or non-technical imaging scientist providing recommendations for the selection of cohort-appropriate GM segmentation software.

## Introduction

Neuroimaging is widely used to investigate both pathological and non-pathological neural phenomena. Given the increasing focus on the reproducibility of MRI findings, it is important that investigators share experiences of the many MRI analysis techniques available in a move toward standardization and optimization of methods. Structural MRI is routinely used to investigate potential differences in brain morphology within both clinical and healthy control groups.

Gray matter (GM) volume is a frequently used measure of brain morphology; it is typically reliable, able to discriminate between healthy controls and clinical groups and fast to calculate (1). It is most often measured using automated software tools that separate GM from other tissue types. Thus, high quality delineation or segmentation of GM, white matter (WM), and cerebrospinal fluid (CSF) is critical in achieving accuracy for all forms of volumetric analyses. There are currently a number of automated tools that can be used for GM segmentation. Methodological comparisons of these tools have focused mainly on built-in automated segmentation software within standard neuroimaging analysis packages including Statistical Parametric Mapping (SPM), FMRIB’s Software Library (FSL), and FreeSurfer or on optimization of a single application (2–10). In short, using these methods on phantom data has shown that both SPM 8 and FSL FAST (version 4.1) are reliable and accurate, whereas FreeSurfer (version 4.5) appears to be highly reliable but not necessarily accurate for measuring GM volume (3), and both SPM 5 and FSL were recommended for GM sensitivity in phantom and control data (9).

Typically, GM segmentation tools have been developed and optimized for use on healthy brains (11) and therefore may not show the same level of accuracy and reliability when used in clinical cohorts. Given the potential challenges associated with performing MRI scans in clinical groups, their scans may be of a lower quality due to increased movement and reduced tissue contrast (12). Klauschen et al. (9), for example, found that GM volume is often underestimated in poor quality images with poor contrast and noise, and overestimated in good quality images, indicating possible bias toward reduced GM in patient populations. Furthermore, greater anatomical variability is likely due to the presence of pathology in clinical cohorts leading to poor segmentation performance in software not designed to deal with pathological abnormalities (11). These key factors could lead to inconsistent findings within clinical neuroimaging studies (7).

There is evidence that some GM segmentation tools are sensitive to volumetric change in clinical populations. SPM 8, SPM 12, FSL 4.1.9, and FreeSurfer 5.1.0, for example, are all sensitive to disease-related change in Alzheimer’s disease, with SPM best for scans with increasing noise (4), but generally appear to perform with greater accuracy for cortical GM (CGM) than subcortical GM, as shown in MS patients (13). SPM, FSL, and FreeSurfer have all shown significant bias in GM measurements when comparing participants with Autism Spectrum Disorder to control participants (7); and SPM can overestimate group differences in healthy elderly participants with atypical anatomy—using a voxel-based technique (14). Although these studies provide some explanation for the difficulty experienced with replication in structural MRI studies, especially in clinical participants, these tools are regularly applied to patient cohorts without optimization for unique brain pathology.

Here, we systematically compare the performance of seven GM segmentation tools firstly in a group of well-phenotyped healthy controls and secondly, in a clinical group to highlight potential issues that may occur when applying standard segmentation tools to patients. We used a group of premanifest Huntington’s disease (PreHD) gene-mutation carriers across different stages of HD (15). HD is a fully penetrant genetic neurodegenerative disease caused by an expanded CAG repeat in the huntingtin gene (16, 17). HD gene-carriers can be identified many years prior to formal motor symptom-based clinical diagnosis enabling tracking of the very earliest signs and symptoms, including neural atrophy. Studies using various analysis techniques, including those implemented in SPM and FSL have shown significant GM change over as little as 12 months in HD (16, 18, 19) likely driven by atrophy in the caudate and putamen, primary regions of neurodegeneration in HD. Regional GM studies focusing on cortical thickness and voxel-based morphometry suggest a pattern of atrophy beginning in occipital and posterior regions and progressing anteriorly as HD progresses (15, 16, 20, 21), while other studies have shown that frontal lobe volume is largely affected across all disease stages in HD (22–24). However, despite numerous studies examining GM volume in HD, it has yet to be established which segmentation tools most accurately and sensitively facilitate the measurement of subtle between-group differences and within-group change.

This guide acts as a primer aimed at the novice imaging analyst or non-technical imaging scientist providing recommendations on how best to select segmentation software for quantification of GM volume, in particular CGM, according to the nature of the cohort of interest. As there is no ground truth regarding cortical degeneration in HD *per se*, and therefore no one measure against which to compare the quality of segmentations, we do not provide a ranking of segmentation tools based on their performance, but instead guidelines on how to select the most appropriate segmentation tool according to the type of dataset being investigated. In addition to commonly used segmentation methods (SPM, FSL, and FreeSurfer), we have included two relatively new tools that have yet to be extensively validated, Advanced Normalization Tools (ANTs) and Multi-Atlas Label Propagation with Expectation–Maximization-based refinement (MALP-EM).

## Materials and Methods

### Participants

Participants were recruited from all four sites (London, Leiden, Paris, and Vancouver) of the observational multisite Track-HD study and attended clinical and MRI scanning sessions at yearly intervals between 2008 and 2011 (15). At the baseline visit, participants were required to have a positive genetic test of ≥40 CAG repeats, a burden of pathology score >250 [as calculated by (CAG-35.5) × age (25)]. The cohort was separated into PreHD and manifest HD participants, with manifest participants having a diagnostic confidence score of 4 or more. The PreHD cohort had a score on the Unified Huntington’s Disease Rating Scale (26)-Total Motor Score (TMS) of ≤5, and was split into two groups based on the median expected years to disease onset (27); those estimated to be more than 10.8 years from disease onset were classified as the PreHD-A group and those less than 10.8 years from estimated onset, PreHD-B. The HD participants had a TMS of ≥7, and were split into two groups using the UHDRS based on their Total Functional Capacity (TFC) scores as stage 1 (HD1:TFC = 11–13) or stage 2 (HD2:TFC = 7–10). The control group was comprised of partners, spouses, and gene-negative siblings of the gene-carriers. Full selection criteria and data collection processes have been published previously (15). For all studies, the local ethical committees gave ethical approval and written informed consent was obtained from each participant according to the Declaration of Helsinki.

One hundred participants from Track-HD were included in the current study: 20 controls, 20 PreHD-A participants, 20 PreHD-B participants, 20 HD1, and 20 HD2 participants. The participants were considered for inclusion according to whether they had repeated (back-to-back) structural scans at baseline of Track-HD (baseline scan A and baseline scan B) and follow-up scans at the 2011 time point. The final participants were randomly selected from those with the correct scans whilst aiming to maintain approximate age matching between the groups.

### Data Acquisition

The 3 T T1-weighted scans were acquired from four scanners. Two were Siemens TIM Trio (London; Paris) and two were Philips Achieva scanners (Leiden and Vancouver). The parameters for Siemens were TR = 2,200 ms, TE = 2.2 ms FOV = 28 cm, matrix size = 256 × 256, 208. For Philips, TR = 7.7 ms, TE = 3.5 ms, FOV = 24 cm, matrix size = 242 × 224, 164. The acquisition was sagittal for whole brain coverage. Slice thickness was 1 mm, with no gap between slices. These acquisition protocols were validated for multisite use (15). All images were visually assessed for quality; specifically, artifacts such as motion, distortion, and poor tissue contrast (IXICO Ltd. and TRACK-HD imaging team, London, UK).

### Data Analysis

T1 scans from baseline (2008) and 3-year follow up (2011) were used for this study. All participants had two scans from the baseline time point, collected consecutively (back-to-back), and one scan from the follow-up time point. All T1-weighted scans were bias-corrected using the non-parametric, non-uniform intensity normalization (N3) method (28), with optimized parameters for 3 T data as outlined in Boyes et al. (29). Scans were then processed following the recommended steps for each software package, as detailed below. This typically involved performing brain extraction and then segmenting the extracted brain into different tissue classes, with one tool (ANTs) also requiring template creation. Following this, volumes for the total GM, CGM, and lobular GM were extracted from the GM segmentations.

#### Software Packages

A number of segmentation algorithms were considered for this investigation, with the final tools based on ease of access, frequency of use within the literature and usability of the software. Software packages selected were

- SPM version 8 Unified Segment (30, 31).
- SPM version 8 New Segment.
- SPM version 12 Segment.
- ANTs Atropos version 2.1.0 (32, 33).
- MALP-EM version 1.2 (34, 35).
- FSL FAST using FSL version 5.0.9 (36).
- FreeSurfer version 5.3.0 (37).

Segmentations were performed in the native (or individual) space in which the scans were acquired. All seven tools include brain extraction either as an additional step prior to segmentation or within the automated GM segmentation pipeline. In this study, the brain extraction procedure provided by each tool was used along with the segmentation method. It should be noted that by not using one common brain extraction method prior to performing segmentation there is additional variability introduced. However, the aim of this study was to examine performance of each segmentation tool based on recommended usage and following the steps many novice are likely to run. Since brain extraction is an inherent part of a number of the tools examined (e.g., SPM, FreeSurfer, MALP-EM) and the other tools include brain extraction algorithms optimized to work with their segmentation procedure (e.g., FSL and ANTs) most users would apply the standard processing for each tool.

### Quality Control

**Figure 1:**
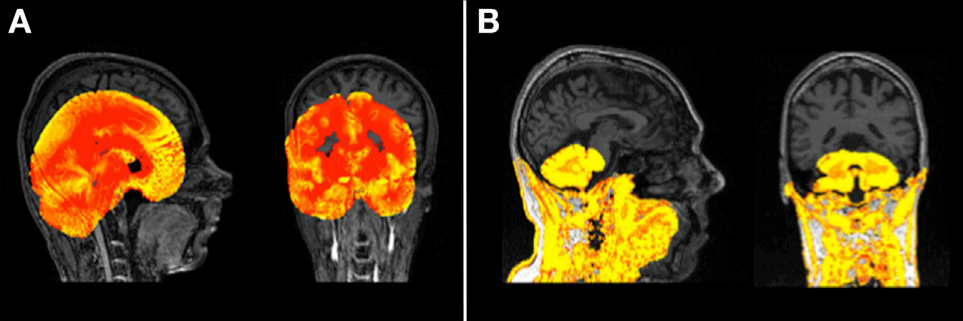
**(A)** An example of a gross failure on a Track-Huntington’s disease (HD) scan when using Statistical Parametric Mapping (SPM) 8 Unified Segment. **(B)** An example of a gross failure on a Track-HD scan when using FMRIB’s Software Library (FSL) Brain Extraction Tool (BET) brain extraction and FAST segmentation procedures.

Following any manipulation of the images scans were visually examined for quality. For this study, processing was classed as a “fail” if there was gross failure in performing the extraction or segmentation (Figure 1), rather than for more minor errors. Gross failures at any stage were initially checked to rule out user error. Processing changes were made to rectify software-based gross errors in two cases. First, SPM 8 Unified Segment delineation of the GM may fail if the orientation of the brain deviates noticeably from the standard SPM templates (Figure 1A). In a few cases, therefore, adjustments were performed to shift the brain orientation to achieve a better match. Second, prior to segmentation using FSL, the Brain Extraction Tool [BET (38)] was run on all data. Using standard parameters BET failed to extract brains satisfactorily on the Track-HD cohort (Figure 1B) and optimized BET parameters were substituted, with these optimized parameters provided in the Supplementary Material.

**Figure 2:**
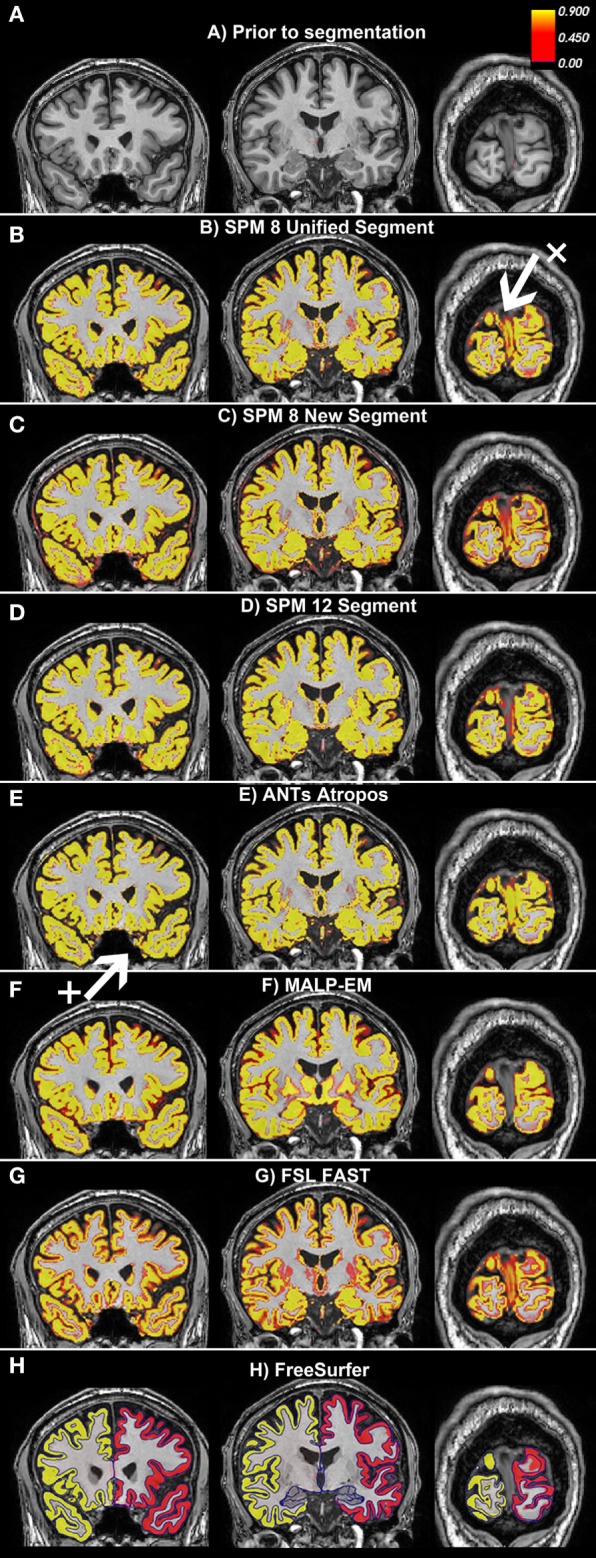
Examples of the gray matter (GM) output from each tool overlaid on one participant from the Track-Huntington’s disease (HD) study. The figure shows three coronal views, with **(A)** showing the same slices with no segmentation. The first slice shows the frontal and temporal regions, the second slice is toward the middle of the brain, and the last slice shows the occipital lobe. All figures show default probabilistic segmentation maps for each software except for FreeSurfer, which shows volumetric and surface-based regions. For the probabilistic segmentation maps, the brighter the yellow within a voxel, the more likely that the voxel contains GM. The arrow labeled x points to a region of the dura incorrectly classified as occipital GM, and the arrow labeled + points to a region of voxels incorrectly classified as temporal GM. **(A)** Prior to segmentation. **(B)** Statistical Parametric Mapping (SPM) 8 Unified Segment, **(C)** SPM 8 New Segment, **(D)** SPM 12 Segment, **(E)** Advanced Normalization Tools (ANTs) Atropos, **(F)** MALP-EM, **(G)** FMRIB’s Software Library (FSL) FAST, and **(H)** FreeSurfer.

During QC, the GM regions were overlaid on the original T1 scans. This enabled easy comparison between the boundaries of the segmented regions and the visible boundaries on the T1 scan. Over- and undersegmentation refers to regions whereby the boundary of the segmented region differed from the visible boundary on the T1. All segmentation tools also showed minor errors as discussed in the results section (Figure 2). No manual intervention was used, however, to avoid adding subjectivity to the measures. Recommendations for manual intervention are provided in the discussion.

### Segmentations

For all GM segmentations except FreeSurfer (see below), volume (ml) was calculated using FSLstats. Probabilistic segmentation maps output by each tool were used in the calculation of volume throughout, as they can account for partial volume effects (PVEs) (39). For each segmentation, total GM, CGM, and lobular GM measures were extracted. Total GM included both CGM and subcortical GM. CGM excluded all subcortical GM structures, and lobular GM divided CGM into the frontal lobe, temporal lobe, parietal lobe, occipital lobe, and insula (see below for further information on extraction of these regions).

Segmentation using all three versions of SPM, FSL, and MALP-EM were run with default settings in native space.

Advanced Normalization Tools brain extraction and segmentation pipeline (Atropos) requires templates and priors. The recommended default pipeline was followed and study-specific templates and priors (32) were created using a subset of 25 Track-HD scans, 5 from each group following the process recommended in Tustison et al. (40). Templates were created for both of the back-to-back baseline datasets and for the final time point using the same participants. Following template creation, ANTs brain extraction was run on each participant using the study-specific template brain mask and priors. The extracted brain for each participant was then used when running the segmentation pipeline, Atropos.

Finally, FreeSurfer was run using the default recon-all pipeline. Unlike the other six segmentation tools, FreeSurfer volumetric maps are not recommended for the calculation of volume as they do not account for PVE. Automatically optimized volumes from the aseg.stats file that are calculated *via* a combination of volumetric and surface-based factors were used for total GM and CGM.^1^

As a number of the segmentation tools do not output CGM and subcortical GM segmentations, for all volumes excluding FreeSurfer a CGM mask was overlaid on total GM to output CGM volumes rather than using each tool’s default regions. While possibly reducing the performance of some tools by using a mask not optimized to that particular technique, this ensured consistency across segmentation techniques.

The Harvard Oxford cortical mask was registered to each participant’s structural scan in native space and used to extract CGM volume from all segmentation outputs (41). As the Harvard Oxford mask includes considerable cerebellar GM, a cerebellum mask was used to exclude cerebellar GM (42). In addition, an MNI mask dividing CGM into five regions (lobes) was also used to examine the performance of the tools within the frontal, temporal, parietal and occipital lobes and the insula. Cortical and lobular masks were binarized and registered to native space *via* ANTs to extract CGM and lobular volumes from the segmentations.

The only exception was FreeSurfer, where total GM and CGM volumes were extracted from an automatically created text file. As FreeSurfer regions have undergone significant optimization during development, we would expect that FreeSurfer may have an advantage over the other tools and this should be considered when exploring the results. To calculate lobular regions for FreeSurfer, the lobular masks for each participant were transformed from native space into each subject’s FreeSurfer analysis space^2^ and used to extract volumes.

### Qualitative Analysis

A qualitative examination of the data was performed initially, as various segmentation issues are best identified by visual examination of the volumetric maps. For example, minor but consistent over- or underestimation of GM within automatically identified anatomical regions may not be detected quantitatively, but can be easily recognized by a trained expert during visual examination. All scans were examined blinded to group and all errors were noted. From this, trends within each tool were detected.

### Quantitative Analysis

Quantitative analysis was performed using Stata version 12.1. Total and CGM volumes were examined in all analyses, and a subset of analyses also examined lobular GM volume. First, summary statistics, including means, ranges, and SDs for demographic information were calculated and between-group differences derived. Total GM, CGM, and lobular GM mean volumes were calculated for both baseline and the follow-up scan for each participant. Additionally, reliability was tested for the back-to-back baseline scans using intraclass correlation (ICC) (43). ICC measures the agreement between repeated ratings, and ranges between 0 and 1 with higher values demonstrating better reliability. Mean repeatability for back-to-back scans was also calculated as percent variability error [Function 1 (40, 44)]. This measure provides a percentage value of variability between repeated applications on the same cohort for each tool; lower values represent less variation. Spearman’s Rho was also used to test the correlation between each set of volumes extracted using each segmentation tool.

*Function 1: Mean repeatability calculation used on back-to-back scans from 2008. Vol_A_ represents the first baseline back-to-back scan, Vol_B_ Represents the second baseline back-to-back scan*:

$$\varepsilon =\frac{\left({\text{Vol}}_{\text{A}}-{\text{Vol}}_{\text{B}}\right)}{0.5\times \left({\text{Vol}}_{\text{A}}+{\text{Vol}}_{\text{B}}\right)}\times 100.$$

To measure the longitudinal sensitivity of each segmentation tool, the control group was compared to all HD groups. Follow-up volume of total GM, CGM, and lobular volumes were expressed as a percentage of baseline volumes and regression analyses were performed to determine whether there were significant differences in the rate of change between controls and each HD group. All results were adjusted for age, gender, and site.

## Results

### Demographics and Clinical Characteristics

Participant demographics are given in Table 1. Age for the two HD groups was slightly higher than for controls and the PreHD groups, but this difference was not significant. CAG was significantly higher in PreHD-B, HD1, and HD2 than in PreHD-A, and significantly higher for HD1 and HD2 than for PreHD-B. As expected, disease burden was also increased in the HD groups.

**Table 1:** Demographics for the participants included in the Track-HD analysis showing means, SDs, and ranges.

	Controls (*N* = 20)	PreHD-A (*N* = 20)	PreHD-B (*N* = 20)	HD1 (*N* = 20)	HD2 (*N* = 20)
Age	48.32 (9.28)	48.48 (6.70)	47.74 (7.72)	49.10 (8.19)	50.14 (8.94)
	30.73–62.97	37.27–59.41	38.11–64.13	31.11–59.63	33.26–62.41

Sex (females)	*N* = 13	*N* = 10	*N* = 12	*N* = 9	*N* = 8

Education	4.05 (1.28)	4.30 (2.27)	4.1 (2.03)	4.2 (1.36)	3.55 (2.32)
	2–6	2–6	2–5	2–6	2–6

CAG	N/A	41 (1.21)	42.35 (1.27)	43.35 (1.90)	43.75 (2.45)
		39–43	40–44	40–47	41–52

Disease burden	N/A	259.80 (29.50)	318.43 (23.99)	372.35 (52.05)	399.15 (70.31)
		171–290.75	267.6–356.03	264.75–472.91	287.31–548.74

### Qualitative Analysis

Based on visual examination, each segmentation technique performed somewhat differently, for example, the boundaries between GM/CSF and GM/WM differed slightly for each tool. Some common errors were also identified across a number of the tools. For example, the occipital lobe was frequently overestimated, with voxels in the occipital dura assigned to GM (see Figure 2, arrow x). In addition, temporal regions were often segmented poorly, with unclear boundaries and oversegmentation outside of the GM (Figure 2, arrow +). Table 2 describes the overall performance of each tool, and Figure 2 shows an example of each tool in one participant from the Track-HD study.

**Table 2:** A description of the performance of each tool, with most common issues outlined.

Software	Performance
SPM 8 Unified Segment	Poor temporal delineation very common. Occipital spillage and underestimation of frontal lobes in a number of scans. 6/400 scans segmented excluded from analysis due to gross failure (1 for 2008 A, 2 for 2008 B non-registered, 2 for 2008 B registered, 1 for 2011).
SPM 8 New Segment	Poor temporal delineation very common. Occipital spillage in a number of scans. Classified voxels in the skull and dura as GM in almost all segmentations. No scans failed segmentation.
ANTs Atropos	Variable performance. Brain extraction determined segmentation quality; e.g., large brain mask meant dura included in the segmentation. Frequent overestimation of occipital GM and poorly delineated temporal lobes. No segmentation fails.
MALP-EM	Fewer issues with overestimation of the occipital lobe. Generally better temporal lobe delineation. CGM underestimated in superior regions in a small number of cases. No segmentation fails.
FSL FAST	Standard BET provided extremely poor brain extractions on Track-HD data and was re-run with an optimized BET procedure, although results of the optimized BET were still subpar. Often underestimated GM volume, with occasional overestimated due to poor brain extraction. As a result, difficult to characterize GM segmentation, and segmentations were deemed as pass or fail, with a fail representing a complete failure to segment the GM. Only two scans were rated as a complete fail.
FreeSurfer	For the volumetric regions, GM tended to be very tight along CSF boundary, with a layer of voxels on the GM/CSF boundary typically excluded from the volume-based segmentation. During the automated partial volume calculation used within FreeSurfer, volume is calculated *via* a combination of the volume- and surface-based segmentations and so some of these excluded voxels would be included in the calculation if they are within the cortical surface. Spillage into the temporal CSF and occipital dura regularly seen with some cases classifying skull as GM.

### Cross-sectional Quantitative Analysis

#### Volumetric Measures

**Figure 3:**
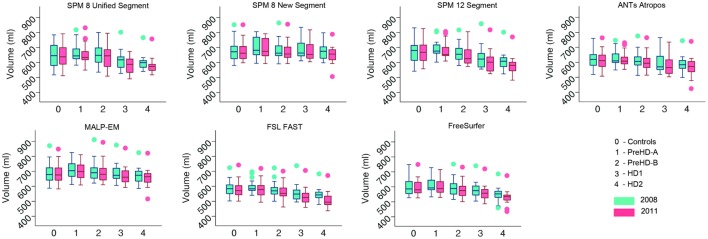
Box plots showing total gray matter (GM) volumes for all groups and all tools for 2008 and 2011 timepoints. Boxes show the first quartile, median, and third quartile, with whiskers representing the smallest and largest value not classified as an outlier. Dots represent outliers.

**Figure 4:**
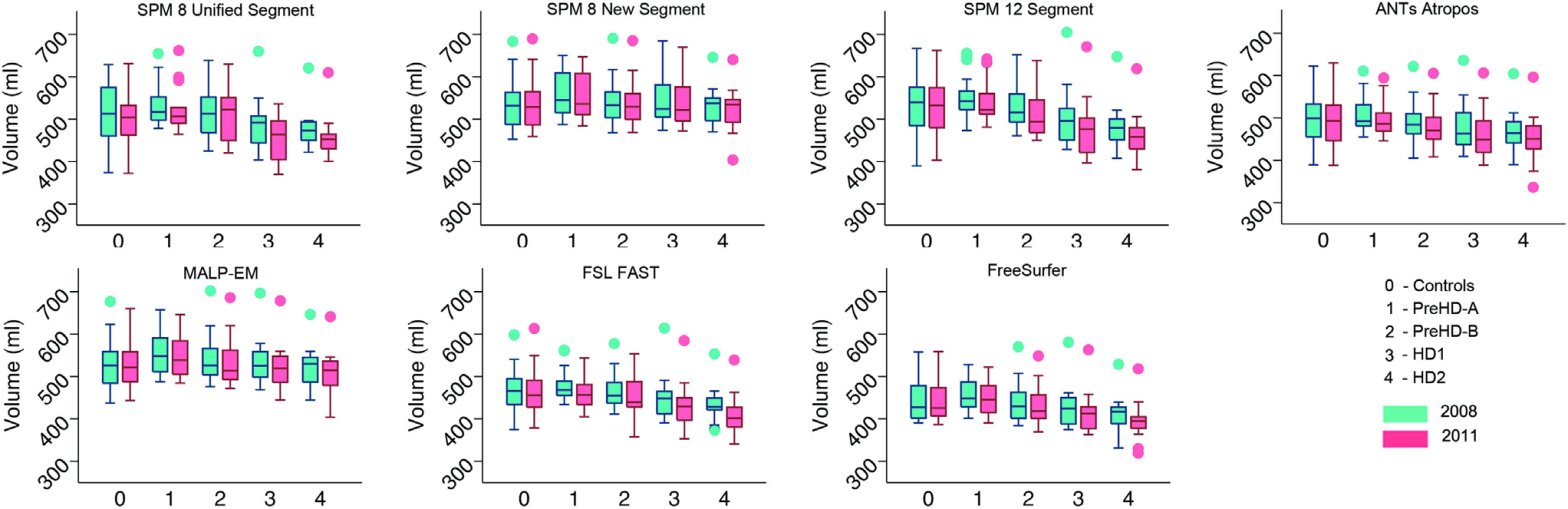
Box plots showing cortical gray matter (GM) volumes for all groups and all tools for 2008 and 2011 timepoints. Boxes show the first quartile, median, and third quartile, with whiskers representing the smallest and largest value not classified as an outlier. Dots represent outliers.

Volumes of the total GM, CGM, and lobular GM were extracted for control participants and participants at different stages of HD. Total GM and CGM volumes are described for both baseline and follow-up time points for each segmentation tool (Figures 3 and 4; Table S1 in Supplementary Material). For all techniques, both total GM and CGM volumes were lower in participants with more advanced disease stage.

For lobular volumes, all tools showed more discrepancies in raw volume (Table 1; Figures S1–S5 in Supplementary Material). For the frontal lobe, all techniques estimated higher GM volume in PreHD-A participants than in controls, with some techniques also estimating higher GM volume in PreHD-B compared to controls. Frontal lobe volume was reduced in HD1 and HD2 groups compared to control and premanifest groups. Temporal lobe volumes were also higher in PreHD-A than in controls for all segmentation tools, with both SPM 12 and MALP-EM estimating higher temporal volume in all groups compared to controls. Other tools showed slightly lower volumes in PreHD-B, HD1, and HD2 than in controls. Parietal lobe volumes showed more uniform volume differences between each technique, with all techniques except for FreeSurfer measuring higher parietal lobe volumes in PreHD-A, PreHD-B, and HD1 compared to controls, and lower volume in HD2 compared to controls. FreeSurfer showed lower volumes in all groups from PreHD-B when compared to controls. For the occipital lobe, the results were variable for each technique, with most techniques showing higher volumes in PreHD-A than controls, and slightly reduced volume with increasing disease progression. Only FreeSurfer showed large reductions in volume between each group. Finally, insula volume was largest in PreHD-B for all techniques except FreeSurfer, with between-group differences appearing minimal for most tools.

#### Reliability Measures

##### Control Participants

For controls, ICC values for baseline scan pairs were above 0.90 for total GM, CGM, and lobular GM volume using each segmentation tool (Table 3; Table S2 in Supplementary Material). Mean repeatability (indexing variability) was lowest in total GM for all techniques, ranging from 0.35% (SPM 8 New Segment) to 1.36% (FreeSurfer), as shown in Table 3, CGM showed only slightly higher variability than total GM for all techniques. Lobular regions generally showed higher repeatability values than total GM and CGM, indicating more variability between the lobular volumes for the first and second back-to-back baseline scans (Table 3).

**Table 3:** (A) Intraclass correlation coefficients and confidence intervals for control participants for back-to-back segmentations of total GM, CGM, frontal lobe GM, temporal lobe GM, parietal lobe GM, occipital lobe GM, and insula GM included in the current study; (B) repeatability values for back-to-back segmentations of total GM, cortical GM, frontal lobe GM, temporal lobe GM, parietal lobe GM, occipital lobe GM, and insula GM for all control participants included in the current study, showing means, SDs, and ranges.

	SPM 8 Unified Segment	SPM 8 New Segment	SPM 12 Segment	ANTs Atropos	MALP-EM	FSL FAST	FreeSurfer
**(A) Intraclass correlations confidence intervals**							

Total GM	0.994	0.999	0.997	0.982	0.998	0.986	0.978
	0.985–0.998	0.997–1.000	0.993–0.999	0.951–0.993	0.995–0.999	0.960–0.995	0.947–0.991

Cortical GM	0.994	0.999	0.997	0.985	0.998	0.988	0.967
	0.985–0.998	0.998–1.000	0.993–0.999	0.958–0.994	0.995–0.999	0.964–0.996	0.918–0.987

Frontal Lobe	0.996	0.999	0.997	0.983	0.997	0.989	0.960
	0.990–0.998	0.997–0.000	0.991–0.999	0.955–0.993	0.993–0.999	0.969–0.996	0.902–0.984

Temporal Lobe	0.989	0.994	0.992	0.990	0.994	0.985	0.975
	0.973–0.996	0.986–0.998	0.980–0.997	0.975–0.996	0.984–0.997	0.963–0.994	0.936–0.990

Parietal Lobe	0.995	0.997	0.996	0.976	0.996	0.984	0.956
	0.986–0.998	0.993–0.999	0.990–0.998	0.931–0.991	0.990–0.998	0.954–0.994	0.886–0.983

Occipital Lobe	0.994	0.993	0.995	0.971	0.992	0.978	0.962
	0.985–0.998	0.984–0.997	0.988–0.998	0.922–0.989	0.981–0.997	0.936–0.992	0.906–0.985

Insula	0.977	0.979	0.979	0.982	0.985	0.979	0.975
	0.941–0.991	0.948–0.992	0.948–0.992	0.955–0.993	0.962–0.994	0.947–0.991	0.938–0.990

**(B) Mean repeatability (SD) range**							

Total GM	1.08 (0.82)	0.35 (0.34)	0.69 (0.48)	1.14 (1.65)	0.41 (0.53)	0.91 (1.33)	1.36 (1.43)
	0.18–2.88	0.01–1.00	0.12–1.77	0.00–6.44	0.02–2.07	0.05–4.99	0.05–5.77

Cortical GM	1.06 (0.82)	0.36 (0.25)	0.79 (0.50)	1.23 (1.71)	0.49 (0.57)	1.14 (1.36)	1.82 (2.09)
	0.02–2.72	0.00–0.98	0.07–1.96	0.03–6.81	0.01–2.18	0.03–4.81	0.06–8.05

Frontal Lobe	1.13 (0.93)	0.50 (0.43)	1.09 (0.67)	1.50 (2.28)	0.67 (0.74)	1.43 (1.49)	2.33 (3.05)
	0.00–3.09	0.05–1.48	0.20–2.45	0.02–9.65	0.00–2.67	0.01–5.72	0.01–11.04

Temporal Lobe	1.50 (2.63)	0.82 (1.52)	1.06 (2.27)	1.25 (1.86)	0.90 (1.71)	1.54 (2.07)	3.16 (2.32)
	0.03–11.93	0.02–7.09	0.04–10.50	0.00–7.27	0.00–7.62	0.02–8.36	0.29–7.46

Parietal Lobe	1.07 (0.88)	0.59 (0.79)	0.88 (0.87)	1.57 (2.35)	0.80 (0.75)	1.29 (1.91)	2.21 (2.79)
	0.01–3.29	0.04–3.72	0.14–3.78	0.01–7.25	0.00–2.74	0.11–6.03	0.10–8.84

Occipital Lobe	1.02 (0.96)	0.85 (0.82)	0.88 (0.63)	1.36 (1.79)	0.75 (0.86)	1.29 (1.63)	2.23 (1.86)
	0.07–3.53	0.04–3.29	0.15–2.51	0.07–6.57	0.07–3.93	0.03–6.00	0.00–6.56

Insula	1.73 (2.42)	1.48 (1.66)	1.44 (2.13)	1.51 (1.76)	1.31 (1.88)	1.58 (2.21)	2.07 (1.91)
	0.08–10.97	0.05–7.91	0.22–9.90	0.21–8.32	0.01–8.44	0.09–10.55	0.09–7.43

Spearman’s Rho correlations showed that there were strong relationships between the volumes extracted using the seven different tools, with values above 0.90 common (Table 4). Correlations were higher for CGM than for total GM. Spearman’s Rho for lobular regions are shown in Tables S4–S8 in Supplementary Material. Relationships >0.75 were seen for all measures for controls across all regions, with most results >0.90, indicating a high level of agreement between tools. Overall, SPM 8 Unified segment showed the lowest relationships with other measures.

**Table 4:** Spearman’s rank correlation for control participants for total GM and CGM.

	SPM 8 Unified Segment	SPM 8 New Segment	SPM 12 Segment	ANTs Atropos	MALP-EM	FSL FAST
**Total GM**						

SPM 8 Unified Segment	1					
SPM 8 New Segment	0.761	1				
SPM 12 Segment	0.904	0.857	1			
ANTs Atropos	0.788	0.920	0.929	1		
MALP-EM	0.812	0.958	0.928	0.967	1	
FSL FAST	0.867	0.896	0.956	0.929	0.944	1
FreeSurfer	0.874	0.904	0.884	0.874	0.905	0.920

**Cortical GM**						

SPM 8 Unified Segment	1					
SPM 8 New Segment	0.798	1				
SPM 12 Segment	0.918	0.971	1			
ANTs Atropos	0.809	0.976	0.934	1		
MALP-EM	0.861	0.932	0.953	0.974	1	
FSL FAST	0.897	0.932	0.947	0.944	0.967	1
FreeSurfer	0.844	0.910	0.893	0.919	0.962	0.956

Differences in ICC and repeatability for different scanner manufactures were briefly examined in control participants. Results for Siemens and Philips scanners were highly comparable for all techniques, and so no further analysis was done to compare the effects of different scanners in this cohort.

##### HD Gene-Carrier Participants

Reliability for total GM, CGM, and lobular GM as measured by ICC was above 0.90 for most tools across all regions and the disease subgroups (Table 5; Table S2 in Supplementary Material). In total GM and CGM, ICC values tended to be lower for PreHD-A than for other HD groups.

**Table 5:** (A) Intraclass correlation coefficients and confidence intervals for HD participants for all tools measuring total GM volume in back-to-back 2008 scans; (B) intraclass correlation coefficients and confidence intervals for HD participants for all tools measuring CGM volume for back-to-back 2008 scans.

	PreHD-A (*N* = 20)	PreHD-B (*N* = 20)	HD1 (*N* = 20)	HD2 (*N* = 20)
**(A) Total GM intraclass correlations confidence intervals**				

SPM 8 Unified	0.993	0.977	0.984	0.989
	0.982–0.997	0.943–0.991	0.960–0.994	0.973–0.996

SPM 8 New Segment	0.999	0.999	0.999	0.999
	0.998–1.000	0.998–1.000	0.998–1.000	0.998–1.000

SPM 12	0.989	0.990	0.997	0.994
	0.972–0.995	0.974–0.996	0.994–0.999	0.982–0.998

Atropos	0.975	0.994	0.989	0.986
	0.912–0.991	0.981–0.998	0.970–0.996	0.938–0.995

MALP-EM	0.996	0.998	0.999	0.996
	0.989–0.998	0.996–0.999	0.996–0.999	0.990–0.998

FAST	0.989	0.997	0.995	0.994
	0.970–0.996	0.989–0.999	0.982–0.998	0.985–0.998

FreeSurfer	0.988	0.988	0.992	0.993
	0.967–0.995	0.968–0.995	0.979–0.997	0.983–0.997

**(B) Cortical GM intraclass correlations confidence intervals**				

SPM 8 Unified	0.993	0.979	0.987	0.991
	0.982–0.997	0.947–0.992	0.967–0.995	0.977–0.996

SPM 8 New Segment	0.999	0.999	0.999	0.999
	0.998–1.000	0.998–1.000	0.998–1.000	0.998–1.000

SPM 12	0.988	0.991	0.998	0.995
	0.971–0.995	0.978–0.996	0.995–0.999	0.984–0.998

Atropos	0.970	0.995	0.991	0.990
	0.893–0.990	0.984–0.998	0.974–0.997	0.947–0.997

MALP-EM	0.995	0.998	0.999	0.996
	0.986–0.998	0.994–0.999	0.997–10.000	0.991–0.999

FAST	0.986	0.996	0.995	0.995
	0.961–0.994	0.989–0.998	0.985–0.998	0.987–0.998

FreeSurfer	0.985	0.983	0.989	0.992
	0.957–0.994	0.955–0.993	0.970–0.996	0.981–0.997

Repeatability values were more variable than ICC values. Repeatability was lower with increasing disease stage for total GM and CGM (Table 6). For individual lobes, it was more variable across disease stages, lobes, and tools, but showed a small range of mean values (Table S3 in Supplementary Material). Values ranged from 0.37% (parietal lobe volume measured by MALP-EM in HD1 participants) to 4.51% (temporal lobe volume measured by FreeSurfer in HD2 participants).

**Table 6:** (A) Repeatability values for back-to-back segmentations of total GM for all HD participants included in the current study, showing means, SDs, and ranges; (B) repeatability values for back-to-back segmentations of CGM for all HD participants included in the current study, showing means, SDs, and ranges.

	SPM 8 Unified	SPM 8 New	SPM 12	Atropos	MALP-EM	FAST	FreeSurfer
**(A) Total GM**							

PreHD-A Total GM	0.89 (0.80)	0.27 (0.19)	0.97 (0.86)	1.26 (1.63)	0.58 (0.53)	0.82 (0.67)	0.90 (0.87)
	0.01–3.05	0.02–0.80	0.01–2.93	0.07–5.61	0.02–2.22	0.00–2.42	0.03–2.69

PreHD-B Total GM	1.61 (1.83)	0.28 (0.22)	0.83 (0.88)	0.78 (0.72)	0.40 (0.40)	0.54 (0.51)	1.15 (0.98)
	0.24–7.03	0.02–0.70	0.02–3.15	0.07–2.45	0.01–1.37	0.02–1.75	0.03–3.85

HD1 Total GM	1.13 (1.33)	0.29 (0.24)	0.64 (0.54)	1.00 (1.27)	0.39 (0.27)	0.82 (0.68)	0.91 (0.74)
	0.04–6.22	0.01–0.75	0.08–1.80	0.02–4.45	0.08–1.20	0.13–2.20	0.15–3.55

HD2 Total GM	0.94 (0.77)	0.22 (0.12)	0.86 (0.71)	1.24 (1.33)	0.57 (0.50)	0.65 (0.70)	0.80 (0.72)
	0.07–2.90	0.03–0.37	0.03–3.20	0.02–5.08	0.01–1.87	0.00–3.06	0.05–2.54

**(B) Cortical GM**							

PreHD-A Cortical GM	0.91 (0.76)	0.33 (0.22)	1.03 (0.89)	1.31 (1.77)	0.67 (0.64)	1.01 (0.70)	1.08 (1.03)
	0.05–2.82	0.04–0.75	0.10–3.32	0.09–6.19	0.05–2.72	0.02–2.41	0.03–3.15

PreHD-B Cortical GM	1.61 (1.90)	0.31 (0.27)	0.86 (0.81)	0.77 (0.69)	0.47 (0.47)	0.65 (0.53)	1.49 (1.28)
	0.09–7.24	0.00–1.10	0.06–2.91	0.15–2.60	0.02–1.70	0.00–1.86	0.23–5.22

HD1 Cortical GM	1.14 (1.44)	0.35 (0.25)	0.67 (0.55)	1.02 (1.09)	0.37 (0.31)	0.87 (0.66)	1.30 (1.11)
	0.11–6.76	0.03–0.88	0.06–1.71	0.00–3.69	0.03–1.43	0.11–2.35	0.27–5.16

HD2 Cortical GM	0.96 (0.78)	0.22 (0.16)	0.86 (0.70)	1.13 (1.11)	0.61 (0.49)	0.66 (0.63)	1.04 (0.96)
	0.04–2.74	0.02–0.61	0.02–3.10	0.04–3.47	0.09–1.85	0.11–2.77	0.04–3.17

For total GM and CGM, Spearman’s correlation between measures tended to be lower for the HD2 group, indicating that the techniques perform differently on more atrophied brains (Tables S4 and S5 in Supplementary Material). SPM 8 Unified Segment again had lower values than other techniques for Spearman’s Rho, especially with SPM 8 New Segment in HD2 participants, whereby correlations of 0.441 and 0.411 were seen for total GM and CGM, respectively. For lobular regions, the relationships between measures were generally lower than those in control participants, with more correlations between 0.7 and 0.9 (Tables S6–S10 in Supplementary Material).

### Longitudinal Quantitative Analysis

#### Total GM Volume

**Figure 5:**
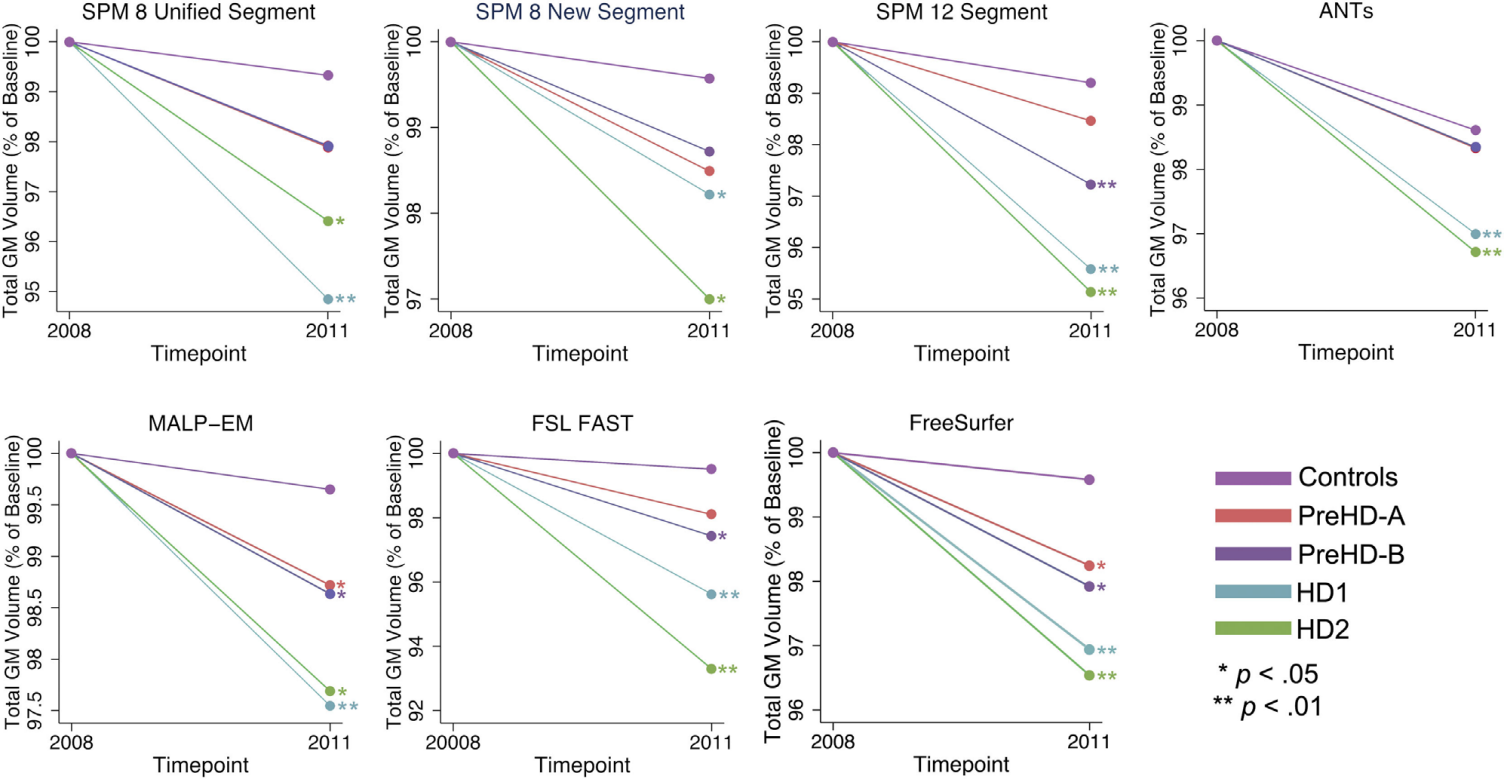
Mean values for all tools and groups showing 2011 volume as a percentage of baseline volume in total gray matter (GM). Significant change difference relative to controls after controlling for age, gender, and site is represented by *p < 0.05, **p < 0.01.

Total GM volume change (as a percentage of baseline volume) for all tools was smaller in controls than that of the HD gene-carrier groups (Table 7; Figure 5). However, when total GM volume change within each HD group was statistically compared to controls, MALP-EM, and FreeSurfer were the only two tools that detected significantly greater change in all disease groups. All other tools detected significantly greater change in HD1 and HD2 compared to controls, with SPM 12 and FAST also showing greater change in PreHD-B compared to controls.

**Table 7:** Mean change (2011 volume as a percentage of baseline volume), SD, and ranges for all tools and groups in total GM.

		Controls	PreHD-A	PreHD-B	HD1	HD2
SPM 8 Unified Segment	% change 2008–2011	−0.67 (3.05)	−2.11 (3.06)	−2.08 (4.53)	−5.15 (6.97)	−3.58 (2.06)
		5.42–7.93	2.13–11.07	7.22–14.38	7.37–30.87	0.53–6.28

	Significant difference	—	−1.12 (0.75–3.00)	−0.62 (0.55–1.79)	−1.71 (0.65–2.76)	−0.53 (0.06–1.00)
			*p* = 0.239	*p* = 0.299	*p* = 0.002	*p* = 0.027

SPM 8 New Segment	% change 2008–2011	−0.43 (2.41)	−1.51 (1.09)	−1.28 (0.76)	−1.78 (1.04)	−3.00 (3.39)
		7.93–3.23	0.32–3.30	0.26–2.60	−0.14–4.12	0.28–16.62

	Significant difference	—	−1.05 (0.05–2.16)	−0.44 (0.07–0.95)	−0.41 (−0.06–0.76)	−0.59 (−0.09–1.08)
			*p* = 0.062	*p* = 0.089	*p* = 0.021	*p* = 0.021

SPM 12 Segment	% change 2008–2011	−0.80 (2.29)	−1.54 (3.61)	−2.77 (1.64)	−4.42 (2.06)	−4.87 (2.50)
		4.79–3.97	−7.49–7.29	0.28–5.89	0.82–7.80	0.98–9.65

	Significant difference	—	−0.43 (1.40–2.26)	−1.00 (−0.42–1.58)	−1.21 (−0.78–1.65)	−0.78 (−0.36–1.19)
			*p* = 0.646	*p* = 0.001	*p* = 0.000	*p* = 0.000

ANTs	% change 2008–2011	−1.39 (2.38)	−1.67 (2.57)	−1.65 (2.18)	−3.00 (1.96)	−3.28 (4.26)
		0.83–9.69	2.79–6.97	2.17–7.17	−0.10–7.09	1.40–18.87

	Significant difference	—	−0.59 (0.90–2.08)	−0.20 (0.44–0.84)	−0.58 (−0.16–1.00)	−0.75 (−0.18–1.31)
			*p* = 0.435	*p* = 0.544	*p* = 0.006	*p* = 0.009

MALP-EM	% change 2008–2011	−0.35 (1.36)	−1.28 (1.29)	−1.37 (1.20)	−2.45 (2.36)	−2.31 (2.72)
		3.81–2.55	1.28–4.68	1.08–3.59	3.34–9.15	1.76–11.83

	Significant difference	—	−0.89 (−0.06–1.72)	−0.47 (−0.10–0.85)	−0.65 (−0.28–1.02)	−0.39 (−0.02–0.75)
			*p* = 0.036	*p* = 0.013	*p* = 0.001	*p* = 0.039

FAST	% change 2008–2011	−0.49 (3.57)	−1.90 (7.83)	−2.57 (3.02)	−4.38 (4.47)	−6.70 (5.81)
		7.91–8.88	26.04–11.48	0.72–12.70	4.55–12.72	−0.32–18.59

	Significant difference	—	−0.30 (–2.62–3.23)	−1.15 (−0.44–1.86)	−1.05 (−0.56–1.55)	−0.85 (−0.37–1.33)
			*p* = 0.839	*p* = 0.002	*p* = 0.000	*p* = 0.001

FreeSurfer	% change 2008–2011	−0.42 (1.90)	−1.75 (1.62)	−2.08 (1.54)	−3.06 (1.70)	−3.46 (1.80)
		−5.96–2.51	−2.15–4.86	−0.97–4.98	−0.16–5.95	0.13–6.30

	Significant difference	—	−1.13 (−0.06–2.21)	−0.87 (−0.37–1.37)	−0.85 (−0.49–1.22)	−0.64 (−0.31–0.97)
			*p* = 0.039	*p* = 0.001	*p* = 0.000	*p* = 0.000

Results of regression analyses comparing rate of change in controls to HD groups, with significantly greater change in HD groups represented by *p < 0.05 (light green), **p < 0.01 (dark green). Age, gender, and site were controlled for.

#### CGM Volume

**Figure 6:**
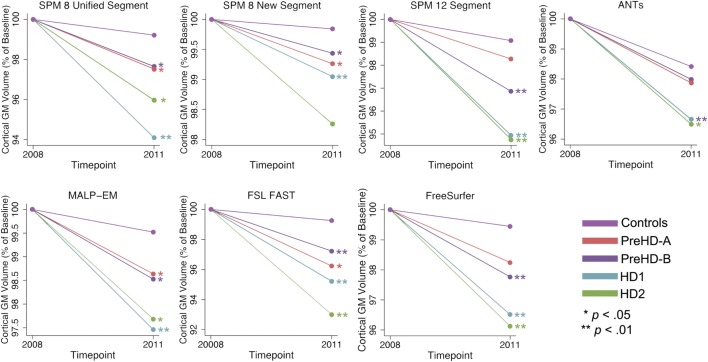
Mean values for all tools and groups showing 2011 volume as a percentage of baseline volume in cortical gray matter (GM). Significant change difference relative to controls after controlling for age, gender, and site are represented by *p < 0.05, **p < 0.01.

The same analysis was conducted in CGM showing that CGM change was inconsistent across tools (Table 8; Figure 6). Except for SPM 8 New Segment, all tools showed significantly greater change in HD1 and HD2 compared to controls, SPM 8 New Segment only showed greater change in HD1. MALP-EM, FAST, and FreeSurfer all showed greater change in PreHD-B than controls, and only MALP-EM and FreeSurfer showed greater change in PreHD-B than controls.

**Table 8:** Mean change (2011 volume as a percentage of baseline volume), SD, and ranges for all tools and groups in cortical GM.

		Controls	PreHD-A	PreHD-B	HD1	HD2
SPM 8 Unified Segment	% change 2008–2011	−0.94 (3.06)	−2.05 (2.17)	−1.73 (3.71)	−4.55 (4.11)	−4.03 (2.30)
		5.17–8.55	2.37–5.99	7.96–8.46	7.45–10.73	0.71–7.24

	Significant difference		−0.89 (0.82–2.59)	−0.40 (−0.63–1.42)	−1.26 (−0.56–1.97)	−0.55 (−0.06–1.03)
			*p* = 0.309	*p* = 0.447	*p* = 0.000	*p* = 0.028

SPM 8 New Segment	% change 2008–2011	−0.16 (0.93)	−0.74 (0.90)	−0.56 (0.62)	−0.95 (0.74)	−1.74 (3.22)
		1.52–1.84	1.21–2.79	0.87–2.26	0.29–2.60	0.82–14.87

	Significant difference		0.56 (1.07–0.06)	0.20 (0.39–0.00)	0.31 (0.46–0.15)	0.29 (0.65–0.07)
			*p* = 0.030	*p* = 0.046	*p* = 0.000	*p* = 0.114

SPM 12 Segment	% change 2008–2011	−0.93 (2.35)	−1.73 (3.87)	−3.14 (1.71)	−5.06 (2.39)	−5.24 (2.61)
		4.68–4.25	7.83–7.98	−0.58–6.16	−1.17–9.06	−1.30–10.43

	Significant difference		0.50 (2.421.42)	1.13 (1.72–0.53)	1.38 (1.86–0.91)	0.83 (1.26–0.40)
			*p* = 0.608	*p* = 0.000	*p* = 0.000	*p* = 0.000

ANTs	% change 2008–2011	−1.58 (2.75)	−2.13 (2.55)	−2.01 (1.90)	−3.34 (1.79)	−3.51 (3.95)
		1.25–11.81	2.21–7.71	2.00–5.84	−0.26–6.61	0.81–17.69

	Significant difference		0.81 (2.440.83)	0.27 (0.95–0.42)	0.64 (1.09–0.18)	0.71 (1.28–0.13)
			*p* = 0.335	*p* = 0.446	*p* = 0.006	*p* = 0.017

MALP-EM	% change 2008–2011	−0.48 (1.24)	−1.36 (1.26)	−1.47 (1.24)	−2.54 (2.69)	−2.32 (2.10)
		3.13–2.49	1.13–4.08	0.73–3.82	3.65–10.92	1.37–9.09

	Significant difference		0.78 (1.55–0.01)	0.45 (0.80–0.09)	0.66 (1.05–0.26)	0.33 (0.62–0.04)
			*p* = 0.047	*p* = 0.013	*p* = 0.001	*p* = 0.027

FAST	% change 2008–2011	−0.74 (3.41)	−3.77 (4.51)	−2.77 (3.55)	−4.78 (4.88)	−7.00 (6.34)
		6.63–8.33	2.62–12.54	0.36–15.00	3.85–14.14	0.09–20.82

	Significant difference		1.96 (3.48–0.45)	1.12 (1.83–0.41)	1.09 (1.59–0.59)	0.85 (1.37–0.33)
			*p* = 0.011	*p* = 0.002	*p* = 0.000	*p* = 0.001

FreeSurfer	% change 2008–2011	−0.55 (1.49)	−1.75 (1.77)	−2.23 (1.86)	−3.49 (2.04)	−3.87 (2.04)
		3.32–2.26	1.94–6.24	1.37–5.69	0.50–6.51	0.13–7.69

	Significant difference		0.98 (1.98–0.03)	0.85 (1.33–0.37)	0.93 (1.28–0.58)	0.70 (1.00–0.40)
			*p* = 0.057	*p* = 0.000	*p* = 0.000	*p* = 0.000

Results of regression analyses comparing rate of change in controls to HD groups, with significantly greater change in HD groups represented by *p < 0.05 (light green), **p < 0.01 (dark green). Age, gender, and site were controlled for.

#### Lobular GM Volume

Longitudinal change within the lobes was variable for all groups with the parietal and occipital lobes showing the most consistent patterns of group differences across most techniques.

##### Frontal Lobe Volume

There was significant frontal lobe change in HD groups compared to controls (Table S11 in Supplementary Material). SPM 8 New Segment and ANTs showed no significantly different change in any group compared to controls. MALP-EM and SPM 8 Unified Segment only detected significant change in HD1 compared to controls, and SPM 12 detected change in PreHD-B, HD1, and HD2 compared to controls. Both FSL FAST and FreeSurfer found significant differences in longitudinal change in all HD groups.

##### Temporal Lobe Volume

For the temporal lobe, SPM 8 New Segment, MALP-EM and ANTs showed no significant differences in volumetric change. FSL FAST and SPM 8 Unified segment found significant differences in HD1 compared to controls, and FreeSurfer and SPM 12 found differences in HD1 and HD2 compared to controls (Table S12 in Supplementary Material).

##### Parietal Lobe Volume

Change in the parietal lobe was more widely detected across groups, with all techniques except SPM 8 Unified Segment showing significantly greater volume reduction in HD1, HD2, and PreHD-B compared to controls, which only showed a difference between HD1 and controls. SPM 8 New Segment, MALP-EM, FAST, and FreeSurfer detected significantly greater change over time in all HD groups (Table S13 in Supplementary Material).

##### Occipital Lobe Volume

Occipital lobe change was again widespread across all tools in HD groups compared to controls (Table S14 in Supplementary Material). SPM 8 New Segment, MALP-EM, FSL FAST, and FreeSurfer found significantly greater change in all HD groups compared to controls. SPM 12 and ANTs found significantly greater change in all PreHD-B, HD1, and HD2 compared to controls and finally SPM 8 Unified Segment found greater change in HD1 and HD2.

##### Insula Volume

Finally, within the insula SPM 8 New Segment and MALP-EM found no differences between any group and controls, SPM 8 Unified Segment, ANTs, FAST, and FreeSurfer found greater change in HD1 and HD2 than controls, and SPM 12 found greater change in PreHD-B, HD1, and HD2 (Table S15 in Supplementary Material).

## Discussion

We compared seven segmentation tools in controls and HD participants. Our aim was to identify the key advantages and disadvantages of each tool and use this information to provide advice for the non-technical user on selecting the most appropriate method to use according to the nature of the cohort. The segmented data were examined qualitatively and quantitatively with a particular focus on CGM. Subcortical changes are already well characterized in HD but there is increasing recognition that the cortex plays an important role prior to disease onset and particularly around the time of conversion to manifest disease (17). Moreover, delineation of the cortex is generally more problematic than subcortical segmentation due to its convoluted structure. Results showed while few segmentations were classified as a “gross” failure following visual examination many tools show poor delineation of the GM especially in temporal and occipital regions, with wide variation in the quality of segmentation performance across tools. This was further evident in the quantitative results, with large differences in segmentation across tools for raw total GM, CGM, and lobular GM volumes. Longitudinal analysis demonstrated that while the pattern of total GM and CGM change was similar across tools, when GM change in HD participants was statistically compared to GM change in controls, the tools detected differing degrees of change. In addition, for lobular volumes the tools showed different patterns of change in a number of regions. Despite the variability in both raw volumes and sensitivity to change, all tools generally showed high reliability across groups and regions, as measured by ICC and repeatability metrics, and extracted volumes were typically highly correlated between different tools.

For controls and HD participants, GM regions derived using MALP-EM were consistently larger than those for other tools, likely due to the regions having higher probabilistic GM segmentation values. SPM also outputted comparatively large regions, with FSL FAST and FreeSurfer outputting the lowest; a discrepancy that has been previously noted (7). SPM regularly overestimated both occipital and temporal lobe regions, this was particularly noticeable for larger brains and in earlier versions of SPM, indicating that SPM may overestimate between-group differences when comparing healthy to atrophied brains. Low volumes using FSL can be explained by regular underestimation of the CGM, partly resulting from poor brain extraction in this cohort. It is important to note that the poor performance of BET should not rule out the use of FSL in other cohorts, since it is known to perform well on other data. Lower volumes output by FreeSurfer likely result from subtle under-estimation seen throughout the cortical boundary with CSF. While the partial volume included *via* calculation of FreeSurfer regions would account for some of these voxels, the regions remained tight along the outer boundary after accounting for this. ANTs showed some evidence of over- and undersegmentation.

Although all tools demonstrated errors using segmentation default pipelines, no manual intervention was performed to improve the quality of segmentation due to the increased subjectivity involved in manual intervention. However, it is important to note that all tools offer some opportunity to improve the issues described above *via* optimization of the segmentation and/or manual intervention after segmentation. For example, FreeSurfer allows editing of the segmented region to improve regions with over- or undersegmentation, MALP-EM, FAST, SPM, and ANTs can all be used with highly optimized masks to improve segmentation, or can be manually edited after segmentation.

Despite the lack of optimization and manual editing it is reassuring to note good reliability for back-to-back scans across tools using ICC and a repeatability metric. These findings support the results of previous studies comparing SPM, FSL, and FreeSurfer (7) and offer additional information on the performance of ANTs and MALP-EM. Most tools were also highly correlated, again supporting previous studies (7), although correlations between the techniques tended to decrease with increasing disease progression indicating divergence of performance on brains with more advanced atrophy. It is likely that using optimized brain masks or performing manual editing may improve performance on atypical brains.

Different techniques showed different longitudinal sensitivity to GM change in HD groups compared to controls, especially within the five lobes. For total GM and CGM, all techniques showed decreasing volume with increasing disease progression. While SPM 8 Unified Segment, SPM 12, and FAST showed the largest decreases in total GM volume over time, MALP-EM and FreeSurfer both showed significant change across all disease stages, possibly indicating greater sensitivity to small changes. In CGM, SPM 8 Unified Segment, SPM 12, and FAST showed large decreases in raw GM volume, but again MALP-EM and FreeSurfer showed statistical sensitivity to small changes. MALP-EM was developed for use in clinical cohorts, which could partly explain the sensitivity of MALP-EM in an HD cohort (35).

The results of the longitudinal lobular analysis show large differences between the tools on each lobe, with particular divergence between tools in the frontal lobe, temporal lobe, and insula. The results of this analysis emphasize the importance of good quality segmentations, with some significant results being driven by participants with very high rates of change. For example, a significantly greater volumetric loss in PreHD-A compared to controls in the frontal lobe as measured by FSL FAST was possibly driven by a percentage loss in one participant of 18% between baseline and follow-up. Re-examination of the segmentations revealed underestimation of the frontal lobe in the follow-up timepoint rather than a true volumetric change. If manual editing had been performed this result would be accounted for, and a more biologically plausible rate of change measured. The impact of segmentation errors is reduced in large cohorts or with whole-brain analyses, but when examining regional change the quality of every slice of the segmentation is paramount. This result supports the work of Iscan et al. (6) who found that by including scans which have poorly delineated FreeSurfer regions in an analysis, the sample size required to detect a true effect increases. It is possible that the results shown in the parietal and occipital lobe are more uniform across tools as this is a region that is thought to undergo the most atrophy within CGM in PreHD and early HD (16, 45–47), and so statistical sensitivity to change in these regions is more robust when segmentation quality is sub-optimal and variability is high. In comparison, regions that may have slower rates of change in HD (e.g., frontal lobe) are more easily biased by poor segmentations and thus different techniques identify different patterns of atrophy. While this study is underpowered to draw conclusions on the true nature of lobular progression of GM atrophy in HD, the results provide a useful demonstration on the importance of selecting a segmentation tool that performs well on a particular dataset.

The results of this study are limited by a few factors. First, the aim was to examine segmentation procedures as they would routinely be applied by non-technical users and thus default brain extraction was used. This introduces additional variability to the comparison, and between-technique differences would likely be reduced if the same brain extraction was used prior to segmentation. In addition, we did not compare the longitudinal pipelines offered by some tools (e.g., FreeSurfer, SPM) as not all currently offer a longitudinal pipeline. Finally, in this study, we used scans from multiple sites which could impact the performance of different techniques. However, this is also a relevant point given the increasing number of multi-site studies.

The results of this study can inform the selection of a GM segmentation tool for use in the Track-HD cohort, but they can also be generalized to other clinical cohorts, particularly neurodegenerative diseases. All tools showed consistently high reliability when used with our clinical data. However, there were a significant number of segmentation errors and while segmentation quality for each tool can be optimized, it is important to note variable results are likely depending on factors including scanner parameters, quality of data and researcher expertise. We now use this analysis as the basis for a set of considerations on how to select of the most appropriate segmentation tool in different types of datasets.

### Which Segmentation Tool Best Answers Your Question?

It is important to consider which software tools contain features that are most appropriate for addressing your question or hypothesis. For example, if regional GM volume is the main focus, considering software that includes the option to perform regional analysis and atlas optimization can potentially reduce errors otherwise introduced when applying a rough mask to your cohort. Some tools (for example, SPM, ANTs, FSL, MALP-EM) are suitable for extracting volumes from customized regions or atlases applied to volumetric maps, while others, such as FreeSurfer, recommend using default atlases. Furthermore, the intended use of other structural analyses such as cortical thickness or VBM should also be considered. More broadly speaking, if T1 weighted imaging is being considered for a study it is important to ensure that it is the most appropriate method for examining the expected clinically relevant pathology, as opposed to techniques for example the use of diffusion weighted imaging would likely be more appropriate for studying WM pathology.

### Are Your Knowledge, Experience, and Resources Sufficient to Use the Tool?

While some tools offer numerous options for customization, this often requires an appropriate level of analysis and computing expertise. Without full understanding of the changes being applied to a pipeline, there is a risk of producing results that are not accurate, reliable or reproducible. Conversely, some tools provide little or no customization, and thus are simple to apply yet may be limited in the range of appropriate applications. If available, use your chosen tool to analyze test data with the same MRI acquisition parameters as those in your study prior to beginning the analysis on a full cohort of participants. This will reduce delays due to errors or complexity of technique when applying it to a large cohort.

As an example, ANTs has previously been validated with impressive results for both registration and cortical thickness using default pipelines in a healthy cohort (40, 48). However, due to the number of possible optimizations for a clinical cohort, such as creation of a study-specific template and priors, an inexperienced user may find using this tool challenging. When applying ANTs to the Track-HD cohort, the large variation in brain sizes within each patient group meant that when using the study-specific brain mask to extract the brain, dura was often erroneously classified as GM. Group-specific templates may have been more suitable here, but this would have required more time and expertise to create and optimize.

Another consideration is the variable nature of processing time necessary for each tool, which can range from 5 min (SPM) to 24 h (FreeSurfer) per brain. In addition, a number of registration or segmentation tools require high levels of processing power and may not run locally on desktop/laptop machines. Access to a high-powered computer cluster or just a single laptop with limited processing resources might determine which tool you choose. Financial costs also warrant consideration. While all tools examined for this case study are freely available for academic purposes, SPM works on a MATLAB platform that is not freely available. In addition, some tools require a license for use in industry settings and if developing methodology for later use within clinical trials, industry costs of these tools should be considered.

### Which Segmentation Tools Are Most Reliable?

A number of studies have previously demonstrated that some of the tools discussed in the current study have high reliability (3, 9). These findings were supported by current results in the Track-HD cohort. Current versions of all tools, including two not previously validated in this way (ANTs Atropos and MALP-EM), demonstrated high reliability. While repeatability was more variable, and lower in early-HD participants, the values were still very good for most tools. It is important to note that in some back-to-back scan pairs, FreeSurfer resulted in large repeatability values indicating that within some segmentations there were large variations in performance for FreeSurfer. For example, for one PreHD-A participant the repeatability within the frontal lobe was 26.01. When the segmentations were re-examined, it was clear that large regions of the temporal lobe had been excluded from the segmentation. This result is concerning in such a widely used tool, and highlights the importance of visual QC and manual editing. While reliability can be established for all tools in this and previous studies, accuracy is more difficult to determine—see section Which Segmentation Tools Are Most Accurate?

### Which Segmentation Tools Are Most Accurate?

While phantom data can be used to examine the accuracy of volumetric measurement tools in healthy models, the results of phantom analyses do not always represent performance when applied to clinical cohorts. In HD, for example, where the pattern of neural change is not well understood it is challenging to define the tool that provides the greatest accuracy. Often, measures are examined in terms of their overlap to examine accuracy—however since all tools showed consistent errors (e.g., oversegmentation in occipital and temporal regions) this could falsely inflate accuracy results. How do we determine which is the most accurate result? Visual quality control was an important factor when assessing accuracy in this study. MALP-EM appeared to be the most visually accurate tool, with SPM 12 also performing well despite some segmentation errors in temporal and occipital regions. By comparing the results of longitudinal change in this sample to other previously published values of longitudinal GM change in larger HD cohorts (measured by various techniques), it appears that a number of techniques overestimate GM change in this small cohort. SPM 8 New Segment, MALP-EM, ANTs, and FreeSurfer produced values in line with previous studies suggesting that they may be able to detect accurate results from a small sample (16, 18). Since inconsistencies between volumetric neuroimaging tools are thought to result in contradictions within clinical neuroimaging research, it is imperative that the accuracy of each tool is visually examined within a cohort to ensure good performance of the tools on a particular cohort.

### Should I Perform Visual QC of My Data?

This is a necessary step in the processing of any data. All registrations, segmentations and masks in this study were visually checked. Errors in processing, complete segmentation failures or patterns in segmentation errors were only detected by viewing the data. While total failure of a tool to segment an image was rare, consistent errors in segmentation were common. In cases where segmentation did fail completely, the volumetric measurements for total GM and CGM were often not out of line with expected values and so may not be detected in a quantitative check of extreme values but would not provide reliable data on pathology or longitudinal sensitivity.

In this study for cases where segmentation was poor but not classified as a fail, the data were included in the final analysis but this resulted in inflated group differences due to outliers, and a larger sample size would be required to detect true effects (6). The process of performing visual QC and rejecting or editing poor quality data is likely to be easier than recruiting and scanning more patients. When investigating GM volume in a cohort with subtle disease effects such as PreHD patients, the benefits of visual QC are likely to be substantial.

In addition to visual QC, manual editing can be performed where appropriate to increase sensitivity. In large cohorts this may be unfeasible; however, as all techniques showed some segmentation errors in the Track-HD cohort and all offer options for manual editing this should be considered. It is particularly important for studies in which subtle group differences or longitudinal change is expected or when subregions are being examined. Manual editing requires the user to have in-depth knowledge of anatomy and a consistent procedure specifying when manual edition should be performed in order to reduce subjectivity.

### How Similar Are Results across the Different Tools?

For control participants, all tools appeared to produce very similar results for both total GM and CGM; with between–technique correlation coefficients generally high, although slightly higher for CGM volume, indicating that techniques show greater variation in subcortical segmentation (Table 3).

However, in HD participants the correlations were more variable, indicating that some techniques appear to detect disease-related change to a greater extent than others. In total GM volume (Table 6), the relationships between techniques were generally lower than in controls. For CGM, correlation coefficients were higher and more stable than for total GM, indicating that measurement of the subcortical GM may be more variable than CGM across different tools. As marked subcortical atrophy in the caudate and putamen is a defining feature of HD, it is unsurprising that the techniques may perform differently when segmenting this region, especially for tools developed on healthy controls. These results suggest that care should be taken when applying techniques in regions of severe atrophy or change, with much more divergence performance apparent in the use of the tools in these circumstances.

## Conclusion

With a multitude of tools available to measure the volume of GM using MRI scans, the selection of the most appropriate tool can be a challenging first step in a research project, and one that may have a marked effect on the results of research. By using seven segmentation tools to analyze GM volume from 100 MRI scans with back-to-back data at multiple time-points in controls and a unique clinical group, we were able to highlight a number of key points related to the measurement of GM volume. Table 9 provides some characteristics of the tools included in this study that can help to guide the initial decision making process in GM volumetric selection.

**Table 9:** Some characteristics of the different software tools included in the current study.

	SPM8 Unified Segment	SPM8 New Segment	SPM 12 Segment	ANTs	MALP-EM	FAST	FreeSurfer
Total GM volume in default pipeline?	Yes	Yes	Yes	Yes	Yes	Yes	Yes
Regional atlas/volume in default pipeline?	No	No	No	Additional Script	Yes	No	Yes
Probabilistic Segmentation default?	Yes	Yes	Yes	Yes	Yes	Yes	No
Discrete Volume Maps default?	No	No	No	Yes	Yes	Yes	No
Easy to manually output regional volumes from maps?	Yes	Yes	Yes	Yes	Yes	Yes	Requires time
Volumes automatically output in text file?	No	No	No	No	Yes	No	Yes
Time to segment (per brain)	5–10 min	5–10 min	5–10 min	1.5–4 h	1.5–4 h	5–10 min	12–24 h
Can be used to process other GM measures? (e.g., cortical thickness)	VBM	VBM	VBM	Cortical thickness	No	VBM	Cortical thickness; gyrification

All methods compared in this work showed high reliability, supporting the results of previous studies and adding new support for the use of ANTs and MALP-EM, which have not been compared in this way previously. For the current cohort, the tools that detected the greatest raw change over a three-year follow-up period were not always the most sensitive to significant change in this period, indicating that higher variability in performance of these tools reduced their sensitivity to subtle disease-related change. Despite this, all tools detected significant longitudinal change in GM when comparing the most advanced patients in the cohort to controls, meaning that all tools are sensitive to more advanced neural pathology across the whole cortex. Subtle regional differences were not detected by all tools, however. One of the most significant findings from this study is the importance of visual quality control of GM segmentations. Poorly segmented and inaccurate data can easily be included in neuroimaging research if visual QC is omitted. While time consuming, the results of research that uses visual quality control will be more sensitive and accurate than if it is not performed.

No one tool is more appropriate for analysis of every type of dataset or cohort. We have identified several key considerations for the selection of the most appropriate GM segmentation tool. The scientific question, level of expertise and available resources are naturally paramount, while comparison of two or three different tools for ease and success of application is recommended and visual quality control essential.

## Track-HD Investigators

C. Campbell, M. Campbell, I. Labuschagne, C. Milchman, J. Stout, Monash University, Melbourne, VIC, Australia; A. Coleman, R. Dar Santos, J. Decolongon, A. Sturrock, University of British Columbia, Vancouver, BC, Canada; C. Jauffret, D. Justo, S. Lehericy, C. Marelli, K. Nigaud, R. Valabrègue, ICM Institute, Paris, France; N. Bechtel, S. Bohlen, R. Reilmann, University of Münster, Münster, Germany; B. Landwehrmeyer, University of Ulm, Ulm, Germany; S. J. A. van den Bogaard, E. M. Dumas, J. van der Grond, E. P. ’t Hart, Leiden University Medical Center, Leiden, *Netherlands*; N. Arran, J. Callaghan, D. Craufurd, C. Stopford, University of Manchester, Manchester, United Kingdom; D. M. Cash, IXICO, London, United Kingdom; H. Crawford, N. C. Fox, G. Owen, N. Z. Hobbs, N. Lahiri, I. Malone, J. Read, M. J. Say, D. Whitehead, E. Wild, University College London, London, United Kingdom; C. Frost, R. Jones, London School of Hygiene and Tropical Medicine, London, United Kingdom; E. Axelson, D. Langbehn, University of Iowa, IA, United States; and S. Queller, C. Campbell, Indiana University, IN, United States.

## Ethics Statement

For all studies, the local ethical committees gave ethical approval and written informed consent was obtained from each participant according to the Declaration of Helsinki.

## Author Contributions

EJ developed and designed the investigation, analyzed the data, and wrote the manuscript. SG contributed to study design and manuscript preparation. HJ contributed to design and analysis for the Track-HD study and contributed to manuscript preparation. AD and BL contributed to study design and data collection for the Track-HD study. RR contributed to study design and data collection for the Track-HD study and contributed to manuscript preparation. GR contributed to manuscript preparation. ST was principle investigator for the Track-HD study and conceptualized and designed Track-HD and contributed to manuscript preparation. RS contributed to study design and data collection for the Track-HD study and also to the study design, analysis, and manuscript preparation for the current investigation.

## Conflict of Interest Statement

The authors declare that the research was conducted in the absence of any commercial or financial relationships that could be construed as a potential conflict of interest.

## Footnotes

^1^https://surfer.nmr.mgh.harvard.edu/fswiki/MorphometryStats.

^2^https://surfer.nmr.mgh.harvard.edu/fswiki/FsAnat-to-NativeAnat.
