# Supplementary material for: Recommendations for the Use of Automated Gray Matter Segmentation Tools: Evidence from Huntington’s Disease
Source: Front Neurol. 2017 Oct 10;8:519. doi: 10.3389/fneur.2017.00519 (PMC5641297; doi:10.3389/fneur.2017.00519)
Supplement: Supplementary file 1 [file Table_1.DOCX]

**Supplementary Information**

**Methodology**

Due to a failure of standardised bet parameters (bet <input> <output>), optimised parameters were used for each site:

1. London and Paris:

bet <input> <output> -f 0.65 -R -c 104 128 128

1. Leiden:

bet <input> <output> -f 0.3 -R -c 82 120 120

1. Vancouver:

bet <input> <output> -f 0.3 -R -c 82 112 112

Table 1: *Total, cortical and lobular GM Volumes for all groups and all tools for 2008 and 2011 timepoints, showing means, standard deviations and ranges.*

|  |  | **Controls (N=20)** | **PreHD-A**  **(N=20)** | **PreHD-B**  **(N=20)** | **HD1**  **(N=20)** | **HD2**  **(N=20)** |
| --- | --- | --- | --- | --- | --- | --- |
| **Total GM** | | | | | | |
| **SPM 8 Unified** | **2008** | 644.72 (77.40) 517.51-784.50 | 667.41 (64.63) 582.46-818.97 | 650.36 (72.40) 535.75-800.05 | 617.39 (60.66) 527.10-802.07 | 598.58 (48.13) 540.66-766.59 |
|  | **2011** | 642.88 (77.28) 511.55-791.30 | 653.41 (67.68) 549.42-831.86 | 637.43 (81.73) 508.94-795.31 | 583.76 (53.99) 491.05-673.19 | 577.33 (51.22) 517.65-757.70 |
| **SPM 8 New Segment** | **2008** | 678.94 (71.25) 580.01-852.75 | 703.62 (66.95) 594.52-805.85 | 683.88 (63.96) 591.00-864.42 | 682.13 (57.10) 611.38-834.77 | 673.07 (47.86) 598.17-798.72 |
|  | **2011** | 675.48 (67.24) 598.10-851.22 | 692.85 (64.90) 592.63-791.93 | 675.12 (63.58) 592.57-855.63 | 669.83 (54.54) 604.48-817.85 | 653.26 (55.78) 506.65-788.99 |
| **SPM 12** | **2008** | 671.50 (72.43) 542.23-831.21 | 691.43 (60.49) 602.55-811.37 | 663.25 (57.93) 579.39-817.00 | 629.56 (72.22) 556.09-857.89 | 607.31 (58.86) 523.90-801.68 |
|  | **2011** | 665.82 (70.42) 557.72-826.72 | 680.32 (59.33) 610.00-806.47 | 645.14 (61.23) 573.01-805.50 | 601.98 (72.38) 525.19-818.94 | 578.07 (61.30) 482.20-771.05 |
| **ANTS** | **2008** | 619.58 (58.45) 519.03-760.16 | 631.63 (54.56) 549.13-746.23 | 610.96 (58.83) 512.86-776.31 | 596.48 (64.76) 519.36-764.84 | 587.03 (53.28) 498.50-745.08 |
|  | **2011** | 611.35 (63.58) 507.70-763.61 | 620.91 (53.98) 553.39-730.49 | 601.04 (61.41) 514.44-765.50 | 578.62 (64.08) 503.29-735.35 | 568.61 (65.08) 424.38-740.74 |
| **MALP-EM** | **2008** | 690.71 (69.12) 587.04-871.31 | 716.21 (62.02) 612.20-825.52 | 699.34 (69.17) 621.59-910.91 | 688.33 (59.38) 606.16-874.72 | 673.63 (52.88) 585.90-824.78 |
|  | **2011** | 687.95 (65.36) 582.47-849.07 | 706.87 (59.69) 608.90-810.98 | 689.91 (70.09) 609.88-893.67 | 671.31 (58.60) 592.95-854.57 | 658.50 (59.41) 516.56-821.83 |
| **FAST** |  | 585.23 (55.70) 502.10-723.89 | 594.94 (43.79) 540.27-695.97 | 577.22 (50.07) 500.41-722.76 | 556.48 (55.90) 495.60-737.27 | 544.08 (42.93) 478.45-684.04 |
|  | **2011** | 582.28 (59.24) 501.16-742.31 | 583.09 (56.16) 493.89-720.33 | 566.74 (54.15) 461.80-701.45 | 543.78 (54.71) 436.44-672.62 | 510.72 (49.95) 436.44-672.62 |
| **FreeSurfer** | **2008** | 600.05 (56.70) 527.34-748.92 | 611.24 (53.11) 533.30-728.35 | 595.52 (57.52) 516.49-752.72 | 576.89 (52.61) 505.51-740.96 | 550.74 (46.71) 461.37-686.31 |
|  | **2011** | 597.41 (56.33) 526.80-749.19 | 600.51 (53.06) 529.69-715.26 | 583.12 (56.77) 501.32-732.18 | 559.12 (50.66) 487.13-719.53 | 531.77 (47.57) 435.12-676.07 |
| **Cortical GM** | | | | | | |
| **SPM 8 Unified** | **2008** | 512.77 (68.98) 373.73-628.56 | 533.18 (51.24) 478.05-654.50 | 516.45 (60.60) 424.98-638.92 | 488.49 (55.33) 403.99-660.65 | 473.58 (41.97) 422.11-620.20 |
|  | **2011** | 505.72 (68.05) 372.24-631.09 | 524.44 (51.07) 464.23-662.46 | 510.59 (65.15) 420.43-629.71 | 460.43 (50.64) 369.75-536.01 | 454.58 (43.52) 400.37-609.44 |
| **SPM 8 New Segment** | **2008** | 536.43 (62.37) 452.89-683.11 | 559.21 (51.87) 487.27-650.20 | 541.97 (52.80) 468.18-690.29 | 539.74 (50.30) 473.83-684.56 | 533.95 (40.36) 469.86-645.25 |
|  | **2011** | 535.67 (63.61) 459.16-689.79 | 555.14 (52.30) 484.16-647.05 | 538.91 (52.49) 468.62-685.50 | 534.52 (48.84) 471.89-669.97 | 525.03 (47.10) 404.13-640.42 |
| **SPM 12** | **2008** | 533.51 (65.41) 389.73-666.73 | 552.73 (49.76) 473.23-655.70 | 526.07 (48.46) 460.87-652.17 | 498.74 (65.09) 428.26-705.08 | 480.93 (50.88) 407.50-647.96 |
|  | **2011** | 528.20 (63.27) 403.20-662.02 | 542.72 (48.25) 481.15-642.66 | 509.88 (51.62) 449.76-638.26 | 473.94 (65.99) 396.61-670.31 | 455.94 (52.03) 380.94-619.42 |
| **ANTS** | **2008** | 495.87 (54.37) 389.28-622.33 | 509.72 (44.12) 455.01-611.42 | 489.45 (48.51) 405.19-620.96 | 477.14 (56.81) 409.16-635.36 | 466.91 (45.54) 389.93-604.35 |
|  | **2011** | 488.38 (58.86) 387.97-630.09 | 498.77 (44.12) 445.98-594.11 | 479.69 (49.48) 408.16-605.48 | 461.18 (55.20) 388.52-606.14 | 451.16 (53.24) 336.37-596.95 |
| **MALP-EM** | **2008** | 530.51 (59.82) 437.13-676.95 | 555.69 (50.24) 487.11-657.42 | 539.48 (55.25) 475.98-702.28 | 533.19 (50.60) 468.57-696.92 | 522.94 (43.59) 444.29-646.02 |
|  | **2011** | 527.67 (56.78) 443.05-660.08 | 547.99 (48.50) 484.60-646.25 | 531.68 (56.27) 471.96-685.57 | 519.58 (50.34) 444.61-678.12 | 511.12 (47.08) 403.89-641.53 |
| **FAST** |  | 469.53 (53.49) 374.92-598.60 | 479.12 (35.49) 434.02-561.92 | 463.95 (41.39) 411.23-577.65 | 447.26 (50.14) 390.53-613.65 | 435.76 (36.32) 374.94-553.17 |
|  | **2011** | 466.03 (56.25) 378.26-613.36 | 460.96 (38.77) 404.72-544.14 | 454.14 (46.04) 357.59-553.83 | 427.99 (51.13) 353.00-584.79 | 408.29 (43.91) 340.86-539.01 |
| **FreeSurfer** | **2008** | 443.98 (46.48) 390.66-557.74 | 456.79 (41.53) 401.60-528.21 | 438.76 (46.32) 383.69-569.80 | 425.94 (48.04) 374.81-581.65 | 409.85 (40.17) 331.48-528.49 |
|  | **2011** | 441.53 (46.83) 386.74-558.92 | 448.86 (42.55) 390.67-522.61 | 428.95 (45.78) 369.63-548.28 | 410.99 (46.52) 362.78-563.30 | 394.04 (40.47) 318.51-518.13 |
| **Frontal lobe volumes** | | | | | | |
| **SPM 8 Unified** | **2008** | 180.98 (28.52) 106.22-230.90 | 189.90 (19.38) 166.41-232.57 | 184.76 (23.46) 151.79-234.20 | 174.68 (20.13) 143.83-241.28 | 170.53 (14.84) 153.66-219.99 |
|  | **2011** | 177.48 (28.29) 107.34-228.77 | 186.98 (18.77) 159.57-233.12 | 182.80 (24.52) 148.94-227.21 | 164.39 (17.52) 133.36-189.41 | 163.63 (15.01) 145.85-216.00 |
| **SPM 8 New Segment** | **2008** | 189.39 (25.88) 137.26-245.25 | 199.62 (20.12) 173.83-231.57 | 193.43 (20.41) 164.85-249.67 | 193.31 (19.55) 169.09-252.14 | 191.08 (14.61) 162.67-230.14 |
|  | **2011** | 188.58 (26.26) 139.07-246.89 | 197.99 (20.37) 170.54-229.86 | 192.02 (20.24) 163.16-245.60 | 191.83 (18.95) 166.05-246.06 | 187.54 (16.33) 150.15-228.48 |
| **SPM 12** | **2008** | 187.68 (27.48) 110.47-235.58 | 196.61 (18.85) 164.94-232.57 | 187.52 (18.47) 163.93-236.62 | 178.26 (23.41) 150.27-257.28 | 172.80 (17.64) 149.93-229.21 |
|  | **2011** | 185.36 (26.89) 115.06-233.49 | 193.05 (17.87) 167.97-226.18 | 181.59 (19.53) 157.27-229.42 | 169.97 (23.42) 135.86-244.01 | 163.97 (17.56) 140.67-219.26 |
| **ANTS** | **2008** | 175.75 (22.96) 113.82-221.10 | 182.93 (16.80) 164.09-216.83 | 175.84 (18.75) 144.59-228.82 | 171.78 (21.20) 142.45-232.90 | 169.39 (16.87) 134.05-216.09 |
|  | **2011** | 172.02 (25.88) 114.78-226.42 | 178.42 (16.72) 155.15-210.80 | 172.64 (18.96) 145.31-220.67 | 166.58 (20.25) 133.86-221.28 | 163.66 (18.72) 127.13-212.46 |
| **MALP-EM** | **2008** | 191.50 (26.06) 127.79-246.67 | 203.47 (19.52) 178.88-239.10 | 197.69 (21.66) 173.33-259.88 | 195.12 (19.63) 167.21-259.26 | 191.73 (15.84) 167.38-233.41 |
|  | **2011** | 189.58 (24.80) 131.21-237.91 | 199.72 (18.69) 175.11-233.90 | 194.38 (21.24) 169.81-247.90 | 190.07 (19.33) 160.37-250.80 | 186.87 (16.63) 154.65-231.24 |
| **FAST** | **2008** | 163.82 (22.16) 106.03-208.91 | 167.73 (13.40) 148.21-199.57 | 163.68 (15.99) 145.72-210.57 | 157.54 (19.32) 130.85-222.20 | 155.82 (13.72) 138.43-194.28 |
|  | **2011** | 162.78 (24.11) 106.91-218.66 | 161.48 (13.90) 138.58-192.57 | 160.58 (17.43) 127.55-199.38 | 152.57 (17.96) 122.21-210.93 | 144.40 (18.81) 102.41-188.63 |
| **FreeSurfer** | **2008** | 164.48 (21.29) 111.40-206.85 | 171.86 (16.65) 147.76-203.70 | 165.86 (19.12) 140.52-218.79 | 162.12 (17.95) 140.99-221.62 | 156.63 (15.08) 130.10-199.35 |
|  | **2011** | 163.60 (21.65) 111.06-209.39 | 168.27 (17.08) 144.17-202.28 | 162.38 (18.21) 140.12-208.54 | 156.95 (16.93) 134.95-212.67 | 151.26 (14.77) 127.40-195.32 |
| **Temporal lobe volumes** | | | | | | |
| **SPM 8 Unified** | **2008** | 120.97 (17.93) 71.48-146.20 | 127.51 (10.36) 112.63-146.96 | 123.21 (14.15) 99.59-153.25 | 116.77 (13.11) 97.60-158.18 | 115.02 (10.21) 104.52-148.72 |
|  | **2011** | 118.98 (18.33) 68.84-146.83 | 125.47 (10.19) 112.77-148.60 | 121.96 (16.18) 100.22-154.86 | 110.31 (11.60) 87.95-124.77 | 110.68 (10.90) 97.95-147.70 |
| **SPM 8 New Segment** | **2008** | 129.44 (16.30) 90.86-161.29 | 136.18 (11.80) 116.62-153.60 | 132.04 (13.21) 114.01-167.99 | 131.30 (12.11) 112.70-165.40 | 131.83 (10.63) 111.46-155.37 |
|  | **2011** | 128.59 (17.20) 87.26-163.60 | 135.57 (11.74) 116.93-153.61 | 131.54 (13.47) 116.55-168.75 | 130.35 (11.77) 112.59-163.60 | 129.96 (12.45) 99.14-156.36 |
| **SPM 12** | **2008** | 127.74 (17.34) 78.29-157.50 | 133.53 (10.37) 119.30-154.39 | 127.45 (12.42) 108.11-158.60 | 121.01 (14.21) 103.23-168.38 | 118.64 (11.60) 102.63-155.45 |
|  | **2011** | 125.99 (17.66) 76.24-157.23 | 131.45 (9.81) 119.97-152.03 | 124.35 (13.39) 108.65-158.07 | 115.89 (14.91) 99.06-163.07 | 112.99 (12.15) 91.59-150.99 |
| **ANTS** | **2008** | 118.96 (15.20) 75.80-148.50 | 124.00 (9.77) 107.60-143.07 | 118.83 (12.36) 99.04-150.52 | 116.12 (12.85) 98.43-154.42 | 114.89 (10.87) 93.56-145.76 |
|  | **2011** | 116.05 (16.90) 72.29-149.85 | 121.32 (9.31) 107.30-139.22 | 116.90 (12.76) 99.72-149.76 | 112.58 (12.72) 95.98-149.51 | 111.12 (13.20) 81.55-146.39 |
| **MALP-EM** | **2008** | 120.61 (15.37) 78.39-150.22 | 127.41 (10.43) 109.65-146.95 | 123.95 (13.56) 108.43-162.17 | 121.95 (12.17) 104.51-160.46 | 121.97 (11.22) 102.86-149.50 |
|  | **2011** | 119.15 (15.51) 75.05-147.63 | 125.74 (10.25) 108.10-144.92 | 122.45 (14.43) 106.35-161.88 | 119.08 (12.57) 96.72-158.21 | 119.39 (12.37) 90.74-149.86 |
| **FAST** | **2008** | 113.51 (14.88) 72.60-144.30 | 117.72 (7.96) 104.82-132.29 | 113.73 (10.31) 101.20-140.26 | 110.45 (11.86) 95.34-150.09 | 108.67 (9.25) 94.83-135.59 |
|  | **2011** | 111.88 (15.48) 70.35-147.91 | 113.33 (9.02) 98.09-130.50 | 112.00 (11.32) 94.81-137.92 | 106.09 (12.87) 81.21-144.88 | 103.72 (8.91) 92.69-134.05 |
| **FreeSurfer** | **2008** | 106.88 (12.25) 73.24-129.97 | 111.19 (8.50) 96.37-124.84 | 106.40 (11.35) 92.06-135.95 | 102.95 (11.84) 89.00-142.54 | 100.99 (9.07) 83.29-127.77 |
|  | **2011** | 105.75 (13.22) 67.72-129.51 | 109.48 (8.05) 97.48-123.75 | 104.75 (11.77) 91.29-135.46 | 99.55 (11.57) 86.87-139.14 | 96.92 (9.22) 77.98-125.87 |
| **Parietal lobe volumes** | | | | | | |
| **SPM 8 Unified** | **2008** | 115.01 (13.95) 89.95-152.64 | 117.89 (15.58) 95.11-146.54 | 121.34 (12.66) 105.21-152.64 | 117.97 (14.25) 97.86-147.60 | 110.74 (12.89) 89.95-144.75 |
|  | **2011** | 116.75 (15.11) 95.26-148.23 | 119.05 (12.57) 102.71-153.81 | 116.13 (14.61) 96.62-144.24 | 104.72 (12.72) 83.10-129.14 | 103.14 (10.00) 88.11-134.68 |
| **SPM 8 New Segment** | **2008** | 124.02 (12.62) 101.06-160.24 | 123.51 (15.65) 101.20-160.24 | 128.21 (12.34) 108.70-147.85 | 124.11 (12.47) 108.55-158.98 | 122.90 (11.91) 105.71-151.24 |
|  | **2011** | 123.11 (12.67) 90.36-162.22 | 123.96 (15.75) 103.50-162.22 | 127.17 (12.40) 107.32-148.16 | 123.34 (12.11) 108.48-157.08 | 121.58 (11.56) 103.84-148.09 |
| **SPM 12** | **2008** | 116.99 (14.45) 86.12-153.77 | 121.34 (14.76) 101.06-153.41 | 124.82 (12.22) 103.00-149.48 | 118.79 (11.36) 104.84-148.29 | 112.15 (15.47) 94.80-153.77 |
|  | **2011** | 113.40 (15.10) 84.14-153.40 | 120.62 (14.26) 99.82-153.40 | 122.57 (11.57) 105.86-147.62 | 114.89 (11.71) 101.51-143.97 | 106.40 (15.68) 89.46-145.79 |
| **ANTS** | **2008** | 111.28 (12.40) 87.01-146.34 | 114.25 (13.21) 96.43-146.34 | 116.18 (11.09) 100.40-140.44 | 111.81 (11.30) 94.34-143.47 | 108.56 (13.48) 90.78-139.49 |
|  | **2011** | 108.72 (12.86) 75.84-148.00 | 113.70 (13.25) 94.40-148.00 | 113.84 (10.84) 99.54-137.42 | 109.06 (11.38) 95.15-138.12 | 104.74 (13.43) 86.88-132.96 |
| **MALP-EM** | **2008** | 128.68 (13.28) 100.96-171.44 | 128.51 (15.76) 107.34-166.99 | 133.19 (12.95) 111.67-157.42 | 129.25 (14.00) 114.14-171.44 | 127.62 (11.97) 112.08-163.43 |
|  | **2011** | 126.98 (13.19) 94.89-166.33 | 128.34 (15.14) 105.49-163.52 | 131.86 (12.51) 110.97-155.82 | 127.44 (13.74) 112.28-166.33 | 124.78 (11.97) 108.64-158.58 |
| **FAST** | **2008** | 105.40 (11.45) 79.20-142.62 | 108.77 (13.33) 92.09-142.62 | 110.26 (9.35) 97.87-131.35 | 106.65 (9.91) 93.53-133.99 | 102.51 (11.85) 86.09-135.15 |
|  | **2011** | 102.59 (12.27) 77.32-147.36 | 108.67 (14.04) 90.60-147.36 | 106.62 (9.55) 91.76-125.41 | 103.91 (10.16) 83.14-126.64 | 98.60 (12.39) 81.72-128.59 |
| **FreeSurfer** | **2008** | 105.34 (12.16) 88.40-133.55 | 106.83 (10.30) 91.71-125.28 | 104.04 (11.30) 90.43-138.07 | 99.33 (11.99) 85.11-133.53 | 95.51 (10.42) 73.41-122.91 |
|  | **2011** | 104.84 (12.06) 88.65-134.08 | 105.29(10.37) 88.88-124.18 | 100.97 (11.16) 85.43-130.83 | 95.56 (11.65) 82.21-129.22 | 91.82 (10.41) 72.02-119.97 |
| **Occipital lobe volumes** | | | | | | |
| **SPM 8 Unified** | **2008** | 81.47 (9.82) 62.78-101.30 | 82.01 (10.19) 69.74-107.25 | 78.28 (8.65) 64.63-94.07 | 74.65 (10.20) 58.66-100.64 | 69.51 (9.31) 56.92-97.25 |
|  | **2011** | 80.94 (9.33) 63.60-101.97 | 80.63 (10.91) 68.29-111.57 | 77.41 (9.57) 63.09-92.09 | 69.83 (10.00) 52.28-90.05 | 66.12 (9.92) 53.65-95.33 |
| **SPM 8 New Segment** | **2008** | 83.36 (8.97) 71.69-103.91 | 83.61 (9.48) 69.29-104.35 | 80.94 (7.14) 67.62-100.31 | 81.02 (8.10) 70.00-101.82 | 78.34 (7.83) 65.28-97.80 |
|  | **2011** | 83.90 (9.05) 72.03-105.30 | 82.83 (9.79) 69.23-102.81 | 80.60 (6.94) 68.49-99.86 | 79.59 (8.07) 69.17-99.05 | 76.59 (9.57) 55.48-97.59 |
| **SPM 12** | **2008** | 84.39 (8.73) 71.15-106.01 | 84.49 (10.05) 71.30-108.76 | 79.53 (6.62) 68.82-94.83 | 75.13 (12.29) 57.08-108.49 | 69.92 (10.88) 55.83-102.09 |
|  | **2011** | 83.82 (8.23) 68.72-103.74 | 82.55 (10.57) 66.99-107.84 | 76.51 (7.48) 65.74-92.72 | 69.92 (12.49) 52.90-101.33 | 65.13 (11.60) 50.50-96.45 |
| **ANTS** | **2008** | 76.52 (6.94) 66.52-94.94 | 75.39 (9.09) 64.84-99.30 | 71.99 (6.79) 56.89-86.68 | 69.77 (10.13) 57.48-94.04 | 66.02 (9.93) 53.06-92.53 |
|  | **2011** | 76.21 (6.87) 65.75-94.43 | 73.96 (9.38) 62.28-94.63 | 69.97 (6.90) 56.71-84.46 | 66.51 (9.72) 53.45-88.89 | 63.19 (11.42) 42.58-91.34 |
| **MALP-EM** | **2008** | 80.33 (8.04) 70.21-100.69 | 80.95 (9.45) 67.11-102.78 | 78.11 (7.29) 66.93-100.00 | 77.90 (8.10) 65.76-100.17 | 74.22 (8.52) 60.68-95.42 |
|  | **2011** | 80.88 (7.80) 70.39-98.78 | 80.03 (9.18) 67.27-100.26 | 76.96 (7.79) 65.25-98.43 | 75.12 (8.06) 64.76-97.58 | 72.12 (9.52) 54.74-94.83 |
| **FAST** | **2008** | 73.46 (7.46) 59.83-92.11 | 72.38 (8.19) 63.11-91.62 | 69.23 (6.11) 60.51-81.93 | 66.32 (9.25) 53.29-91.62 | 62.13 (8.40) 49.91-85.26 |
|  | **2011** | 72.92 (7.86) 60.31-91.98 | 68.72 (9.47) 49.13-87.01 | 66.89 (8.31) 41.90-79.72 | 60.66 (11.95) 31.04-86.52 | 55.42 (12.33) 34.97-82.97 |
| **FreeSurfer** | **2008** | 66.30 (7.74) 57.48-85.09 | 66.07 (7.47) 56.14-83.26 | 61.89 (6.19) 52.82-76.74 | 59.68 (8.89) 45.74-83.49 | 54.85 (8.43) 42.19-76.40 |
|  | **2011** | 66.26 (7.12) 56.46-83.60 | 65.00 (7.87) 54.31-81.47 | 60.02 (6.45) 49.60-72.51 | 57.10 (8.36) 45.10-80.61 | 52.23 (8.95) 40.04-74.45 |
| **Insula lobe volumes** | | | | | | |
| **SPM 8 Unified** | **2008** | 13.55 (1.63) 10.43-16.92 | 14.54 (1.31) 12.37-17.08 | 14.01 (1.51) 11.55-16.87 | 13.43 (1.65) 11.07-17.64 | 13.20 (1.55) 10.14-17.92 |
|  | **2011** | 13.33 (1.52) 9.83-16.70 | 14.39 (1.28) 12.18-16.66 | 13.82 (1.61) 11.69-16.18 | 12.69 (1.52) 10.31-15.13 | 12.66 (1.64) 9.65-17.62 |
| **SPM 8 New Segment** | **2008** | 13.88 (1.42) 12.01-17.52 | 15.00 (1.57) 12.59-17.77 | 14.38 (1.49) 11.76-17.43 | 14.45 (1.66) 11.80-17.96 | 14.34 (1.56) 11.35-18.34 |
|  | **2011** | 13.98 (1.41) 11.96-17.68 | 14.97 (1.52) 12.53-17.68 | 14.29 (1.43) 12.11-17.29 | 14.41 (1.59) 12.11-17.66 | 14.15 (1.72) 9.98-18.20 |
| **SPM 12** | **2008** | 14.47 (1.61) 11.65-18.40 | 15.32 (1.26) 13.12-17.96 | 14.53 (1.45) 12.18-17.26 | 13.78 (1.86) 11.21-18.97 | 13.55 (1.75) 10.08-19.07 |
|  | **2011** | 14.48 (1.55) 11.94-18.23 | 15.08 (1.30) 12.85-17.46 | 14.15 (1.43) 12.06-16.86 | 13.17 (1.82) 10.83-17.98 | 12.85 (1.88) 9.57-18.43 |
| **ANTS** | **2008** | 12.69 (1.40) 10.49-15.98 | 13.69 (1.27) 11.25-16.18 | 13.06 (1.40) 10.54-15.45 | 12.97 (1.66) 10.35-16.69 | 12.89 (1.42) 10.12-17.09 |
|  | **2011** | 12.71 (1.37) 10.02-15.97 | 13.66 (1.26) 11.19-15.93 | 12.99 (1.32) 10.92-15.18 | 12.72 (1.59) 10.38-16.05 | 12.50 (1.65) 9.06-16.89 |
| **MALP-EM** | **2008** | 12.76 (1.62) 10.76-15.90 | 14.19 (1.82) 11.85-17.57 | 13.63 (1.50) 11.18-15.92 | 13.89 (1.52) 11.12-16.98 | 13.62 (1.58) 10.60-16.90 |
|  | **2011** | 12.93 (1.52) 10.86-15.92 | 14.03 (1.82) 10.61-17.32 | 13.47 (1.51) 11.40-15.78 | 13.73 (1.44) 11.23-16.67 | 13.41 (1.57) 10.24-16.71 |
| **FAST** | **2008** | 12.33 (1.43) 9.90-15.43 | 13.26 (1.15) 11.10-15.33 | 12.70 (1.13) 10.95-14.74 | 12.49 (1.51) 10.20-16.45 | 12.30 (1.40) 9.24-16.22 |
|  | **2011** | 12.39 (1.35) 10.19-15.20 | 13.00 (1.23) 11.15-15.44 | 12.56 (1.06) 10.81-14.15 | 12.17 (1.44) 10.12-15.76 | 11.95 (1.37) 8.67-15.78 |
| **FreeSurfer** | **2008** | 10.91 (1.26) 9.49-13.84 | 11.63 (1.23) 9.52-13.85 | 11.19 (1.16) 9.21-13.08 | 11.26 (1.26) 9.55-13.98 | 11.03 (1.32) 8.18-14.33 |
|  | **2011** | 10.98 (1.28) 9.48-13.85 | 11.48 (1.33) 9.61-13.79 | 11.05 (1.04) 9.59-12.77 | 11.08 (1.15) 9.27-13.67 | 10.65 (1.31) 7.98-14.20 |

*
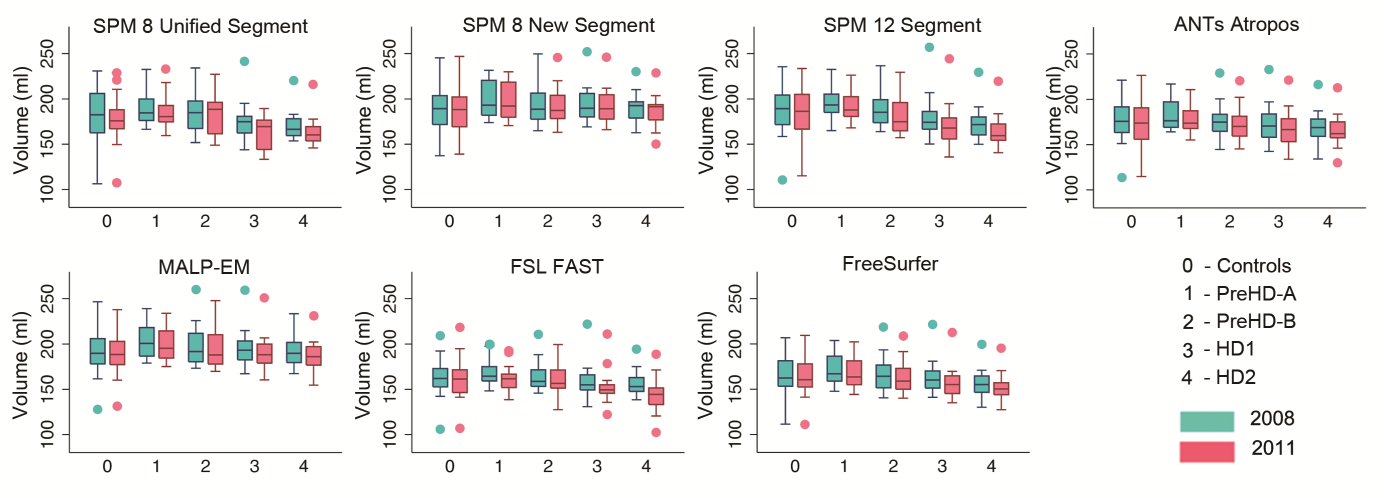
*

*Figure 1:* *Box plots showing frontal GM Volumes for all groups and all tools for 2008 and 2011 timepoints. Boxes show the first quartile, median and third quartile, with whiskers representing the smallest and largest value not classified as an outlier. Dots represent outliers.*

*
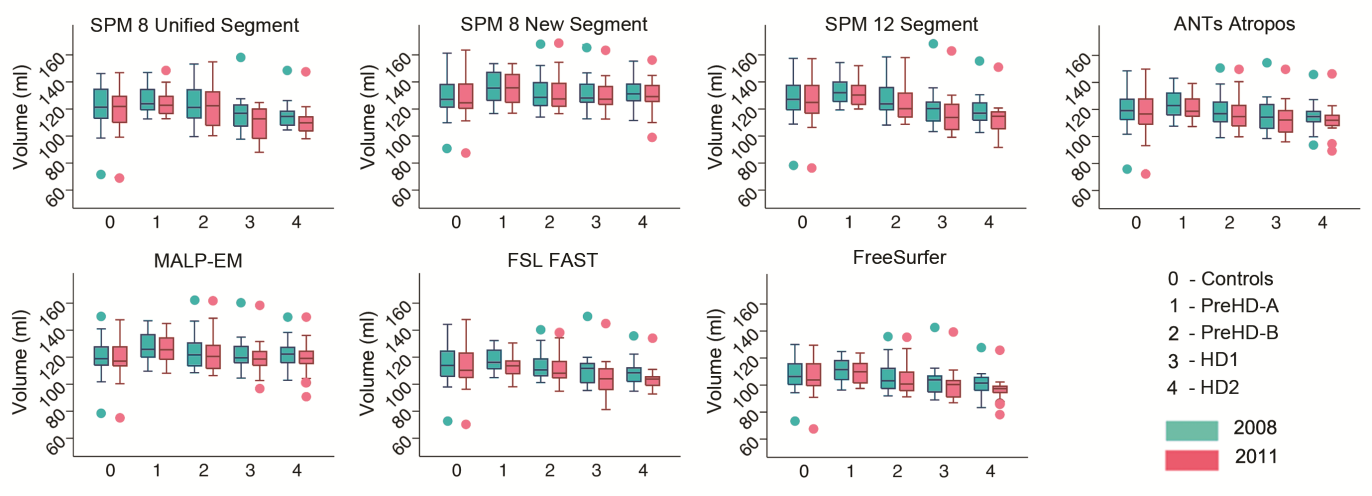
*

*Figure 2:* *Box plots showing temporal GM Volumes for all groups and all tools for 2008 and 2011 timepoints. Boxes show the first quartile, median and third quartile, with whiskers representing the smallest and largest value not classified as an outlier. Dots represent outliers.*

*
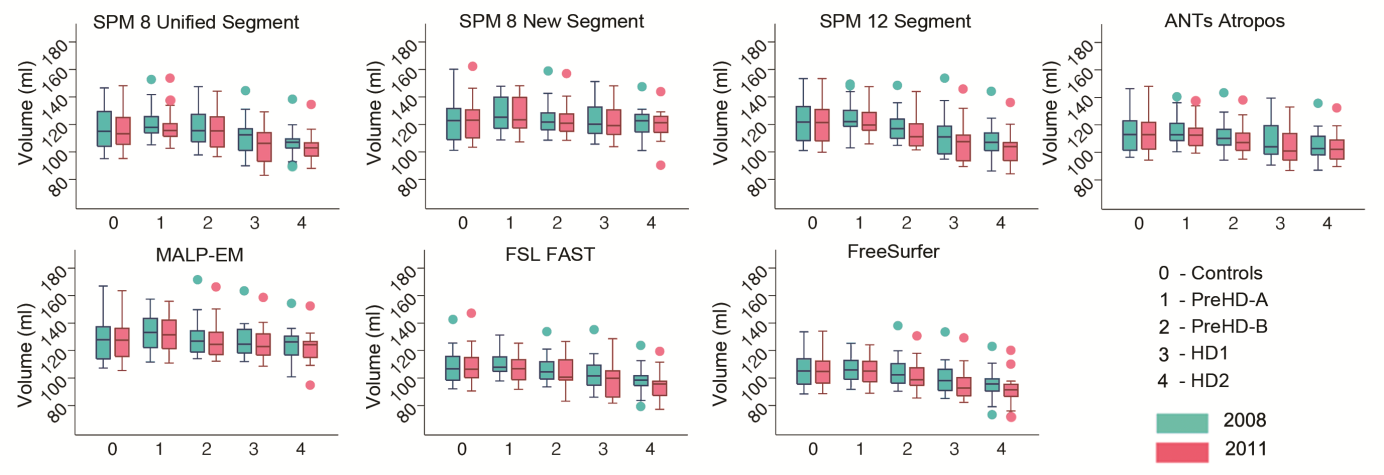
*

*Figure 3:* *Box plots showing parietal GM Volumes for all groups and all tools for 2008 and 2011 timepoints. Boxes show the first quartile, median and third quartile, with whiskers representing the smallest and largest value not classified as an outlier. Dots represent outliers.*

*
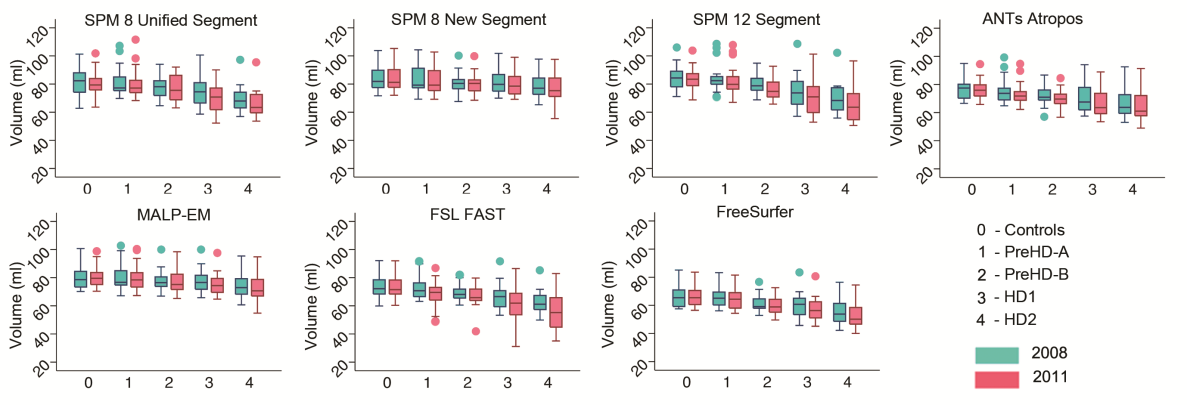
*

*Figure 4:* *Box plots showing occipital GM Volumes for all groups and all tools for 2008 and 2011 timepoints. Boxes show the first quartile, median and third quartile, with whiskers representing the smallest and largest value not classified as an outlier. Dots represent outliers.*

*
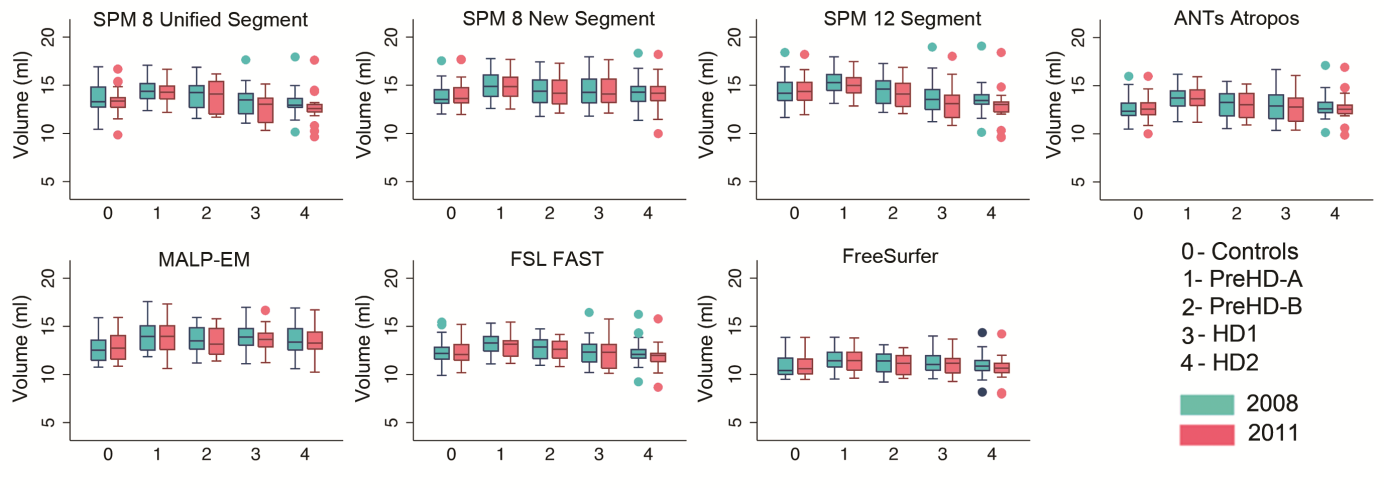
*

*Figure 5:* *Box plots showing insula GM Volumes for all groups and all tools for 2008 and 2011 timepoints. Boxes show the first quartile, median and third quartile, with whiskers representing the smallest and largest value not classified as an outlier. Dots represent outliers.*

*Table 2: Intraclass correlations and confidence intervals for all HD groups in frontal, temporal, parietal, occipital lobes and the insula in different tools.*

|  | **PreHD-A**  **(N=20)** | **PreHD-B**  **(N=20)** | **HD1**  **(N=20)** | **HD2**  **(N=20)** |
| --- | --- | --- | --- | --- |
| 1. **Frontal GM**   **Intraclass Correlations**  **Confidence intervals** | | | | |
| **SPM 8 Unified** | 0.991  0.977-0.997 | 0.979  0.945-0.992 | 0.986  0.967-0.995 | 0.990  0.976-0.996 |
| **SPM 8 New Segment** | 0.997  0.994-0.999 | 0.999  0.994-1.000 | 0.999  0.997-1.000 | 0.998  0.994-0.999 |
| **SPM 12** | 0.987  0.969-0.995 | 0.992  0.981-0.997 | 0.998  0.994-0.999 | 0.994  0.983-0.998 |
| **Atropos** | 0.957  0.868-0.984 | 0.995  0.987-0.998 | 0.990  0.970-0.996 | 0.989  0.952-0.997 |
| **MALP-EM** | 0.992  0.981-0.997 | 0.998  0.995-0.999 | 0.998  0.996-0.999 | 0.996  0.990-0.998 |
| **FAST** | 0.983  0.959-0.993 | 0.993  0.984-0.997 | 0.995  0.981-0.998 | 0.991  0.977-0.996 |
| **FreeSurfer** | 0.973  0.927-0.990 | 0.977  0.942-0.991 | 0.984  0.949-0.994 | 0.988  0.971-0.995 |
| 1. **Temporal GM**   **Intraclass Correlations**  **Confidence intervals** | | | | |
| **SPM 8 Unified** | 0.992  0.979-0.997 | 0.979  0.947-0.992 | 0.990  0.976-0.996 | 0.993  0.982-0.997 |
| **SPM 8 New Segment** | 0.999  0.996-0.999 | 0.999  0.998-1.000 | 0.998  0.996-0.999 | 0.998  0.996-0.999 |
| **SPM 12** | 0.987  0.965-0.995 | 0.992  0.979-0.997 | 0.997  0.992-0.999 | 0.995  0.988-0.998 |
| **Atropos** | 0.959  0.847-0.986 | 0.994  0.983-0.998 | 0.995  0.985-0.998 | 0.991  0.959-0.997 |
| **MALP-EM** | 0.991  0.962-0.997 | 0.998  0.995-0.999 | 0.997  0.993-0.999 | 0.994  0.984-0.997 |
| **FAST** | 0.970  0.903-0.989 | 0.993  0.981-0.997 | 0.996  0.991-0.999 | 0.994  0.986-0.998 |
| **FreeSurfer** | 0.959  0.894-0.984 | 0.979  0.945-0.992 | 0.995  0.987-0.998 | 0.987  0.968-0.995 |
| 1. **Parietal GM**   **Intraclass Correlations**  **Confidence intervals** | | | | |
| **SPM 8 Unified** | 0.994  0.984-0.998 | 0.984  0.959-0.994 | 0.988  0.970-0.995 | 0.990  0.976-0.996 |
| **SPM 8 New Segment** | 0.999  0.997-1.000 | 0.994  0.986-0.998 | 0.998  0.995-0.999 | 0.998  0.995-0.999 |
| **SPM 12** | 0.988  0.970-0.995 | 0.992  0.981-0.997 | 0.998  0.994-0.999 | 0.994  0.985-0.998 |
| **Atropos** | 0.987  0.952-0.996 | 0.992  0.978-0.997 | 0.989  0.968-0.996 | 0.986  0.948-0.995 |
| **MALP-EM** | 0.997  0.993-0.999 | 0.994  0.984-0.998 | 0.999  0.997-1.000 | 0.996  0.989-0.998 |
| **FAST** | 0.989  0.973-0.996 | 0.992  0.980-0.997 | 0.990  0.971-0.996 | 0.992  0.981-0.997 |
| **FreeSurfer** | 0.991  0.977-0.996 | 0.978  0.936-0.992 | 0.981  0.945-0.993 | 0.987  0.967-0.995 |
| 1. **Occipital GM**   **Intraclass Correlations**  **Confidence intervals** | | | | |
| **SPM 8 Unified** | 0.993  0.982-0.997 | 0.968  0.920-0.988 | 0.983  0.958-0.993 | 0.991  0.978-0.996 |
| **SPM 8 New Segment** | 0.999  0.997-1.000 | 0.993  0.983-0.997 | 0.998  0.994-0.999 | 0.996  0.991-0.999 |
| **SPM 12** | 0.990  0.976-0.996 | 0.973  0.934-0.989 | 0.998  0.994-0.999 | 0.993  0.979-0.997 |
| **Atropos** | 0.984  0.947-0.994 | 0.978  0.913-0.993 | 0.991  0.977-0.996 | 0.995  0.967-0.998 |
| **MALP-EM** | 0.994  0.985-0.998 | 0.991  0.974-0.996 | 0.994  0.985-0.998 | 0.996  0.991-0.999 |
| **FAST** | 0.988  0.971-0.995 | 0.990  0.958-0.997 | 0.995  0.986-0.998 | 0.997  0.992-0.999 |
| **FreeSurfer** | 0.987  0.967-0.995 | 0.968  0.908-0.988 | 0.989  0.973-0.996 | 0.995  0.988-0.981 |
| 1. **Insula**   **Intraclass Correlation**  **Confidence Intervals** | | | | |
| **SPM 8 Unified** | 0.990  0.974-0.996 | 0.984  0.960-0.994 | 0.994  0.984-0.997 | 0.994  0.984-0.998 |
| **SPM 8 New Segment** | 0.995  0.988-0.998 | 0.996  0.991-0.999 | 0.998  0.996-0.999 | 0.998  0.995-0.999 |
| **SPM 12** | 0.988  0.969-0.995 | 0.987  0.968-0.995 | 0.997  0.993-0.999 | 0.996  0.989-0.998 |
| **Atropos** | 0.988  0.956-0.996 | 0.993  0.984-0.997 | 0.995  0.987-0.998 | 0.993  0.981-0.997 |
| **MALP-EM** | 0.996  0.990-0.999 | 0.998  0.994-0.999 | 0.997  0.992-0.999 | 0.996  0.990-0.998 |
| **FAST** | 0.992  0.973-0.997 | 0.995  0.988-0.998 | 0.997  0.993-0.999 | 0.997  0.992-0.999 |
| **FreeSurfer** | 0.984  0.960-0.994 | 0.970  0.925-0.988 | 0.964  0.906-0.986 | 0.963  0.910-0.985) |

*Table 3: Repeatability values for back-to-back segmentations of frontal, temporal, parietal, occipital and insula GM for all HD participants included in the current study, showing means, standard deviations, and ranges.*

|  | **PreHD-A**  **(N=20)** | **PreHD-B**  **(N=20)** | **HD1**  **(N=20)** | | **HD2**  **(N=20)** |  |
| --- | --- | --- | --- | --- | --- | --- |
| 1. **Frontal**   **Mean repeatability (Standard Deviation)**  **Range** | | | | | |  |
| **SPM 8 Unified Segment** | 1.06 (0.88)  0.14-3.59 | 1.68 (2.09)  0.05-8.02 | | 1.11 (1.60)  0.11-7.42 | 0.99 (0.78)  0.03-2.76 |  |
| **SPM 8 New Segment** | 0.55 (0.45)  0.01-1.44 | 0.45 (0.32)  0.00-1.09 | | 0.40 (0.25)  0.05-0.94 | 0.44 (0.33)  0.04-1.30 |  |
| **SPM 12** | 1.12 (1.07)  0.08-4.27 | 0.89 (0.78)  0.04-2.35 | | 0.71 (0.60)  0.00-1.93 | 0.91 (0.69)  0.20-3.05 |  |
| **Atropos** | 1.72 (2.37)  0.05-9.03 | 0.84 (0.64)  0.08-2.48 | | 1.27 (1.18)  0.04-4.02 | 1.22 (1.13)  0.02-4.16 |  |
| **MALP-EM** | 0.76 (0.93)  0.00-3.92 | 0.56 (0.39)  0.02-1.33 | | 0.43 (0.37)  0.01-1.58 | 0.57 (0.60)  0.01-2.01 |  |
| **FAST** | 1.22 (0.80)  0.16-2.95 | 0.93 (0.67)  0.06-2.43 | | 0.97 (0.77)  0.19-2.78 | 0.93 (0.87)  0.03-2.69 |  |
| **FreeSurfer** | 1.43 (1.71)  0.00-5.29 | 3.21 (5.54)  0.20-26.01 | | 1.59 (1.35)  0.02-5.51 | 1.28 (1.02)  0.10-3.43 |  |
| 1. **Temporal**   **Mean repeatability (Standard Deviation)**  **Range** | | | | | |  |
| **SPM 8 Unified Segment** | 0.80 (0.76)  0.02-2.66 | 1.49 (1.92)  0.17-7.06 | | 1.11 (1.05)  0.19-4.97 | 0.85 (0.72)  0.03-2.57 |  |
| **SPM 8 New Segment** | 0.37 (0.26)  0.03-0.96 | 0.34 (0.29)  0.03-0.86 | | 0.38 (0.34)  0.04-1.33 | 0.36 (0.33)  0.03-1.08 |  |
| **SPM 12** | 0.98 (0.74)  0.10-2.81 | 0.81 (0.84)  0.06-3.22 | | 0.74 (0.58)  0.06-2.38 | 0.81 (0.55)  0.10-2.01 |  |
| **Atropos** | 1.43 (1.68)  0.17-5.97 | 0.73 (0.79)  0.03-2.85 | | 0.75 (0.78)  0.03-3.33 | 1.07 (1.03)  0.15-3.61 |  |
| **MALP-EM** | 0.77 (0.77)  0.01-2.97 | 0.48 (0.40)  0.00-1.67 | | 0.52 (0.60)  0.00-2.64 | 0.87 (0.78)  0.07-2.36 |  |
| **FAST** | 1.02 (1.20)  0.03-5.12 | 0.75 (0.78)  0.03-2.95 | | 0.69 (0.58)  0.02-2.13 | 0.69 (0.77)  0.02-3.26 |  |
| **FreeSurfer** | 3.15 (2.30)  0.18-7.87 | 3.04 (3.51)  0.18-15.82 | | 3.43 (2.33)  0.26-8.30 | 4.51 (2.81)  0.09-8.49 |  |
| 1. **Parietal**   **Mean repeatability (Standard Deviation)**  **Range** | | | | | |  |
| **SPM 8 Unified Segment** | 0.89 (0.83)  0.22-3.39 | 1.57 (1.64)  0.02-6.21 | | 1.15 (1.37)  0.01-6.38 | 1.04 (0.81)  0.04-2.66 |  |
| **SPM 8 New Segment** | 0.38 (0.33)  0.02-1.49 | 0.54 (0.82)  0.00-3.79 | | 0.46 (0.37)  0.00-1.44 | 0.39 (0.39)  0.00-1.33 |  |
| **SPM 12** | 1.10 (1.02)  0.01-3.89 | 0.84 (0.78)  0.04-2.83 | | 0.71 (0.65)  0.05-2.42 | 0.90 (0.73)  0.07-3.40 |  |
| **Atropos** | 1.08 (1.12)  0.10-4.16 | 0.98 (0.91)  0.04-3.15 | | 1.22 (1.26)  0.07-4.43 | 1.35 (1.45)  0.09-5.60 |  |
| **MALP-EM** | 0.50 (0.53)  0.05-2.32 | 0.72 (0.89)  0.03-3.79 | | 0.37 (0.30)  0.02-1.29 | 0.72 (0.43)  0.14-1.73 |  |
| **FAST** | 0.95 (0.75)  0.09-2.50 | 0.94 (0.71)  0.03-2.43 | | 1.31 (1.04)  0.12-3.14 | 0.82 (0.90)  0.05-3.66 |  |
| **FreeSurfer** | 0.90 (0.83)  0.00-2.63 | 1.99 (1.72)  0.40-7.01 | | 1.66 (1.70)  0.02-6.43 | 1.32 (1.43)  0.01-4.14 |  |
| 1. **Occiptial**   **Mean repeatability (Standard Deviation)**  **Range** | | | | | |  |
| **SPM 8 Unified Segment** | 1.00 (1.24)  0.25-5.84 | 2.03 (2.01)  0.04-7.31 | | 1.55 (1.84)  0.05-8.44 | 1.38 (1.20)  0.10-3.91 |  |
| **SPM 8 New Segment** | 0.52 (0.31)  0.02-1.07 | 0.82 (0.61)  0.01-2.36 | | 0.58 (0.44)  0.01-1.45 | 0.67 (0.60)  0.02-2.10 |  |
| **SPM 12** | 1.27 (1.02)  0.03-4.33 | 1.34 (1.32)  0.00-4.65 | | 0.90 (0.71)  0.02-2.18 | 1.49 (1.20)  0.32-4.53 |  |
| **Atropos** | 1.56 (1.68)  0.15-6.95 | 1.44 (1.40)  0.02-6.44 | | 1.19 (1.27)  0.00-4.47 | 1.25 (1.15)  0.07-4.49 |  |
| **MALP-EM** | 1.05 (0.67)  0.05-2.82 | 0.98 (0.91)  0.02-3.99 | | 0.84 (0.73)  0.03-2.91 | 0.80 (0.72)  0.06-2.42 |  |
| **FAST** | 1.39 (1.00)  0.14-4.15 | 0.94 (0.80)  0.10-2.57 | | 1.17 (0.73)  0.13-2.63 | 0.86 (0.79)  0.11-3.72 |  |
| **FreeSurfer** | 1.47 (1.24)  0.17-4.96 | 2.25 (2.02)  0.13-7.06 | | 1.59 (1.35)  0.09-5.90 | 1.14 (1.13)  0.09-4.54 |  |
| 1. **Insula**   **Mean repeatability (Standard Deviation)**  **Range** | | | | | |  |
| **SPM 8 Unified Segment** | 0.82 (1.00)  0.01-3.21 | 1.43 (1.36)  0.06-4.45 | | 1.02 (0.99)  0.01-4.53 | 1.03 (0.93)  0.08-3.39 |  |
| **SPM 8 New Segment** | 0.81 (0.63)  0.02-2.08 | 0.80 (0.44)  0.09-1.56 | | 0.60 (0.38)  0.02-1.33 | 0.53 (0.43)  0.08-1.37 |  |
| **SPM 12** | 0.86 (0.95)  0.11-3.88 | 1.08 (1.04)  0.09-4.00 | | 0.75 (0.67)  0.04-2.65 | 0.97 (0.84)  0.02-3.36 |  |
| **Atropos** | 1.26 (0.73)  0.08-2.37 | 0.77 (0.90)  0.02-3.73 | | 1.02 (0.72)  0.02-2.82 | 1.08 (0.92)  0.09-3.13 |  |
| **MALP-EM** | 0.89 (0.65)  0.00-2.09 | 0.59 (0.44)  0.01-1.39 | | 0.74 (0.48)  0.02-1.92 | 0.92 (0.60)  0.01-1.79 |  |
| **FAST** | 0.95 (0.63)  0.07-2.10 | 0.69 (0.48)  0.07-1.90 | | 0.72 (0.56)  0.05-1.87 | 0.71 (0.64)  0.10-1.93 |  |
| **FreeSurfer** | 1.64 (1.05)  0.26-4.08 | 2.72 (2.88)  0.34-13.31 | | 1.95 (2.26)  0.03-7.95 | 2.62 (2.27)  0.15-7.77 |  |

*Table 4: Spearman’s ranked correlation for segmentations of total GM for all HD participants included in the current study.*

|  | **SPM 8 Unified Segment** | **SPM 8 New Segment** | **SPM 12 Segment** | **ANTs Atropos** | **MALP-EM** | **FSL FAST** |
| --- | --- | --- | --- | --- | --- | --- |
| **PreHD-A** | | | | | | |
| **SPM 8 Unified Segment** | 1 |  |  |  |  |  |
| **SPM 8 New Segment** | 0.777 | 1 |  |  |  |  |
| **SPM 12 Segment** | 0.896 | 0.666 | 1 |  |  |  |
| **ANTs Atropos** | 0.732 | 0.833 | 0.737 | 1 |  |  |
| **MALP-EM** | 0.808 | 0.950 | 0.749 | 0.844 | 1 |  |
| **FSL FAST** | 0.741 | 0.785 | 0.770 | 0.926 | 0.874 | 1 |
| **FreeSurfer** | 0.865 | 0.959 | 0.752 | 0.805 | 0.952 | 0.802 |
| **PreHD-B** | | | | | | |
| **SPM 8 Unified Segment** | 1 |  |  |  |  |  |
| **SPM 8 New Segment** | 0.770 | 1 |  |  |  |  |
| **SPM 12 Segment** | 0.932 | 0.889 | 1 |  |  |  |
| **ANTs Atropos** | 0.726 | 0.934 | 0.851 | 1 |  |  |
| **MALP-EM** | 0.842 | 0.964 | 0.941 | 0.919 | 1 |  |
| **FSL FAST** | 0.883 | 0.848 | 0.917 | 0.857 | 0.878 | 1 |
| **FreeSurfer** | 0.871 | 0.925 | 0.952 | 0.838 | 0.938 | 0.887 |
| **HD1** | | | | | | |
| **SPM 8 Unified Segment** | 1 |  |  |  |  |  |
| **SPM 8 New Segment** | 0.737 | 1 |  |  |  |  |
| **SPM 12 Segment** | 0.857 | 0.672 | 1 |  |  |  |
| **ANTs Atropos** | 0.847 | 0.818 | 0.779 | 1 |  |  |
| **MALP-EM** | 0.793 | 0.955 | 0.702 | 0.886 | 1 |  |
| **FSL FAST** | 0.845 | 0.731 | 0.815 | 0.917 | 0.841 | 1 |
| **FreeSurfer** | 0.884 | 0.857 | 0.875 | 0.829 | 0.895 | 0.826 |
| **HD2** | | | | | | |
| **SPM 8 Unified Segment** | 1 |  |  |  |  |  |
| **SPM 8 New Segment** | 0.441 | 1 |  |  |  |  |
| **SPM 12 Segment** | 0.758 | 0.555 | 1 |  |  |  |
| **ANTs Atropos** | 0.644 | 0.820 | 0.671 | 1 |  |  |
| **MALP-EM** | 0.633 | 0.917 | 0.719 | 0.860 | 1 |  |
| **FSL FAST** | 0.827 | 0.738 | 0.768 | 0.908 | 0.853 | 1 |
| **FreeSurfer** | 0.605 | 0.802 | 0.796 | 0.681 | 0.883 | 0.726 |

*Table 5: Spearman’s ranked correlation for segmentations of cortical GM for all HD participants included in the current study.*

|  | **SPM 8 Unified Segment** | **SPM 8 New Segment** | **SPM 12 Segment** | **ANTs Atropos** | **MALP-EM** | **FSL FAST** |
| --- | --- | --- | --- | --- | --- | --- |
| **PreHD-A** | | | | | | |
| **SPM 8 Unified Segment** | 1 |  |  |  |  |  |
| **SPM 8 New Segment** | 0.815 | 1 |  |  |  |  |
| **SPM 12 Segment** | 0.883 | 0.722 | 1 |  |  |  |
| **ANTs Atropos** | 0.865 | 0.901 | 0.844 | 1 |  |  |
| **MALP-EM** | 0.836 | 0.970 | 0.755 | 0.886 | 1 |  |
| **FSL FAST** | 0.848 | 0.848 | 0.847 | 0.920 | 0.874 | 1 |
| **FreeSurfer** | 0.868 | 0.955 | 0.811 | 0.917 | 0.973 | 0.923 |
| **PreHD-B** | | | | | | |
| **SPM 8 Unified Segment** | 1 |  |  |  |  |  |
| **SPM 8 New Segment** | 0.747 | 1 |  |  |  |  |
| **SPM 12 Segment** | 0.926 | 0.863 | 1 |  |  |  |
| **ANTs Atropos** | 0.762 | 0.943 | 0.896 | 1 |  |  |
| **MALP-EM** | 0.830 | 0.974 | 0.922 | 0.947 | 1 |  |
| **FSL FAST** | 0.818 | 0.934 | 0.925 | 0.916 | 0.955 | 1 |
| **FreeSurfer** | 0.887 | 0.836 | 0.935 | 0.848 | 0.874 | 0.925 |
| **HD1** | | | | | | |
| **SPM 8 Unified Segment** | 1 |  |  |  |  |  |
| **SPM 8 New Segment** | 0.734 | 1 |  |  |  |  |
| **SPM 12 Segment** | 0.868 | 0.716 | 1 |  |  |  |
| **ANTs Atropos** | 0.869 | 0.898 | 0.844 | 1 |  |  |
| **MALP-EM** | 0.735 | 0.973 | 0.711 | 0.914 | 1 |  |
| **FSL FAST** | 0.820 | 0.764 | 0.862 | 0.902 | 0.782 | 1 |
| **FreeSurfer** | 0.820 | 0.893 | 0.898 | 0.910 | 0.901 | 0.854 |
| **HD2** | | | | | | |
| **SPM 8 Unified Segment** | 1 |  |  |  |  |  |
| **SPM 8 New Segment** | 0.411 | 1 |  |  |  |  |
| **SPM 12 Segment** | 0.826 | 0.576 | 1 |  |  |  |
| **ANTs Atropos** | 0.692 | 0.814 | 0.741 | 1 |  |  |
| **MALP-EM** | 0.638 | 0.910 | 0.749 | 0.869 | 1 |  |
| **FSL FAST** | 0.803 | 0.699 | 0.835 | 0.919 | 0.860 | 1 |
| **FreeSurfer** | 0.729 | 0.729 | 0.887 | 0.832 | 0.868 | 0.880 |

*Table 6: Spearman’s ranked correlation for segmentations of frontal GM for all HD groups included in the current study.*

|  | **SPM 8 Unified Segment** | **SPM 8 New Segment** | **SPM 12 Segment** | **ANTs Atropos** | **MALP-EM** | **FSL FAST** |
| --- | --- | --- | --- | --- | --- | --- |
| **Controls** | | | | | | |
| **SPM 8 Unified Segment** | 1.000 |  |  |  |  |  |
| **SPM 8 New Segment** | 0.807 | 1.000 |  |  |  |  |
| **SPM 12 Segment** | 0.918 | 0.935 | 1.000 |  |  |  |
| **ANTs Atropos** | 0.800 | 0.972 | 0.951 | 1.000 |  |  |
| **MALP-EM** | 0.860 | 0.975 | 0.953 | 0.954 | 1.000 |  |
| **FSL FAST** | 0.891 | 0.879 | 0.940 | 0.907 | 0.925 | 1.000 |
| **FreeSurfer** | 0.863 | 0.940 | 0.914 | 0.900 | 0.965 | 0.902 |
| **PreHD-A** | | | | | | |
| **SPM 8 Unified Segment** | 1.000 |  |  |  |  |  |
| **SPM 8 New Segment** | 0.811 | 1.000 |  |  |  |  |
| **SPM 12 Segment** | 0.919 | 0.749 | 1.000 |  |  |  |
| **ANTs Atropos** | 0.839 | 0.865 | 0.859 | 1.000 |  |  |
| **MALP-EM** | 0.832 | 0.962 | 0.737 | 0.841 | 1.000 |  |
| **FSL FAST** | 0.820 | 0.638 | 0.802 | 0.776 | 0.692 | 1.000 |
| **FreeSurfer** | 0.835 | 0.925 | 0.758 | 0.878 | 0.943 | 0.788 |
| **PreHD-B** | | | | | | |
| **SPM 8 Unified Segment** | 1.000 |  |  |  |  |  |
| **SPM 8 New Segment** | 0.761 | 1.000 |  |  |  |  |
| **SPM 12 Segment** | 0.902 | 0.926 | 1.000 |  |  |  |
| **ANTs Atropos** | 0.770 | 0.971 | 0.935 | 1.000 |  |  |
| **MALP-EM** | 0.821 | 0.968 | 0.949 | 0.943 | 1.000 |  |
| **FSL FAST** | 0.856 | 0.830 | 0.892 | 0.824 | 0.847 | 1.000 |
| **FreeSurfer** | 0.826 | 0.950 | 0.949 | 0.932 | 0.958 | 0.917 |
| **HD1** | | | | | | |
| **SPM 8 Unified Segment** | 1.000 |  |  |  |  |  |
| **SPM 8 New Segment** | 0.806 | 1.000 |  |  |  |  |
| **SPM 12 Segment** | 0.818 | 0.689 | 1.000 |  |  |  |
| **ANTs Atropos** | 0.851 | 0.914 | 0.803 | 1.000 |  |  |
| **MALP-EM** | 0.761 | 0.976 | 0.630 | 0.908 | 1.000 |  |
| **FSL FAST** | 0.678 | 0.713 | 0.677 | 0.803 | 0.713 | 1.000 |
| **FreeSurfer** | 0.875 | 0.896 | 0.868 | 0.925 | 0.875 | 0.762 |
| **HD2** | | | | | | |
| **SPM 8 Unified Segment** | 1.000 |  |  |  |  |  |
| **SPM 8 New Segment** | 0.582 | 1.000 |  |  |  |  |
| **SPM 12 Segment** | 0.869 | 0.704 | 1.000 |  |  |  |
| **ANTs Atropos** | 0.738 | 0.805 | 0.791 | 1.000 |  |  |
| **MALP-EM** | 0.662 | 0.970 | 0.777 | 0.881 | 1.000 |  |
| **FSL FAST** | 0.839 | 0.681 | 0.788 | 0.877 | 0.784 | 1.000 |
| **FreeSurfer** | 0.738 | 0.889 | 0.893 | 0.818 | 0.916 | 0.777 |

*Table 7: Spearman’s ranked correlation for segmentations of temporal GM for all HD participants included in the current study.*

|  | **SPM 8 Unified Segment** | **SPM 8 New Segment** | **SPM 12 Segment** | **ANTs Atropos** | **MALP-EM** | **FSL FAST** |
| --- | --- | --- | --- | --- | --- | --- |
| **Controls** | | | | | | |
| **SPM 8 Unified Segment** | 1.000 |  |  |  |  |  |
| **SPM 8 New Segment** | 0.863 | 1.000 |  |  |  |  |
| **SPM 12 Segment** | 0.972 | 0.932 | 1.000 |  |  |  |
| **ANTs Atropos** | 0.861 | 0.972 | 0.930 | 1.000 |  |  |
| **MALP-EM** | 0.874 | 0.967 | 0.949 | 0.981 | 1.000 |  |
| **FSL FAST** | 0.928 | 0.947 | 0.954 | 0.944 | 0.937 | 1.000 |
| **FreeSurfer** | 0.918 | 0.956 | 0.951 | 0.956 | 0.939 | 0.975 |
| **PreHD-A** | | | | | | |
| **SPM 8 Unified Segment** | 1.000 |  |  |  |  |  |
| **SPM 8 New Segment** | 0.853 | 1.000 |  |  |  |  |
| **SPM 12 Segment** | 0.865 | 0.826 | 1.000 |  |  |  |
| **ANTs Atropos** | 0.908 | 0.944 | 0.856 | 1.000 |  |  |
| **MALP-EM** | 0.844 | 0.937 | 0.818 | 0.964 | 1.000 |  |
| **FSL FAST** | 0.914 | 0.908 | 0.890 | 0.970 | 0.943 | 1.000 |
| **FreeSurfer** | 0.904 | 0.949 | 0.878 | 0.952 | 0.944 | 0.946 |
| **PreHD-B** | | | | | | |
| **SPM 8 Unified Segment** | 1.000 |  |  |  |  |  |
| **SPM 8 New Segment** | 0.767 | 1.000 |  |  |  |  |
| **SPM 12 Segment** | 0.941 | 0.901 | 1.000 |  |  |  |
| **ANTs Atropos** | 0.800 | 0.990 | 0.926 | 1.000 |  |  |
| **MALP-EM** | 0.824 | 0.971 | 0.934 | 0.970 | 1.000 |  |
| **FSL FAST** | 0.908 | 0.895 | 0.962 | 0.899 | 0.925 | 1.000 |
| **FreeSurfer** | 0.896 | 0.896 | 0.956 | 0.890 | 0.916 | 0.974 |
| **HD1** | | | | | | |
| **SPM 8 Unified Segment** | 1.000 |  |  |  |  |  |
| **SPM 8 New Segment** | 0.768 | 1.000 |  |  |  |  |
| **SPM 12 Segment** | 0.892 | 0.689 | 1.000 |  |  |  |
| **ANTs Atropos** | 0.842 | 0.868 | 0.874 | 1.000 |  |  |
| **MALP-EM** | 0.830 | 0.943 | 0.737 | 0.895 | 1.000 |  |
| **FSL FAST** | 0.820 | 0.773 | 0.785 | 0.821 | 0.863 | 1.000 |
| **FreeSurfer** | 0.821 | 0.723 | 0.889 | 0.833 | 0.809 | 0.874 |
| **HD2** | | | | | | |
| **SPM 8 Unified Segment** | 1.000 |  |  |  |  |  |
| **SPM 8 New Segment** | 0.641 | 1.000 |  |  |  |  |
| **SPM 12 Segment** | 0.886 | 0.749 | 1.000 |  |  |  |
| **ANTs Atropos** | 0.779 | 0.829 | 0.839 | 1.000 |  |  |
| **MALP-EM** | 0.770 | 0.925 | 0.865 | 0.935 | 1.000 |  |
| **FSL FAST** | 0.794 | 0.827 | 0.922 | 0.844 | 0.863 | 1.000 |
| **FreeSurfer** | 0.746 | 0.893 | 0.907 | 0.866 | 0.926 | 0.926 |

*Table 8: Spearman’s ranked correlation for segmentations of parietal GM for all HD participants included in the current study.*

|  | **SPM 8 Unified Segment** | **SPM 8 New Segment** | **SPM 12 Segment** | **ANTs Atropos** | **MALP-EM** | **FSL FAST** |
| --- | --- | --- | --- | --- | --- | --- |
| **Controls** | | | | | | |
| **SPM 8 Unified Segment** | 1.000 |  |  |  |  |  |
| **SPM 8 New Segment** | 0.791 | 1.000 |  |  |  |  |
| **SPM 12 Segment** | 0.925 | 0.879 | 1.000 |  |  |  |
| **ANTs Atropos** | 0.854 | 0.947 | 0.942 | 1.000 |  |  |
| **MALP-EM** | 0.833 | 0.981 | 0.877 | 0.951 | 1.000 |  |
| **FSL FAST** | 0.846 | 0.937 | 0.905 | 0.912 | 0.932 | 1.000 |
| **FreeSurfer** | 0.898 | 0.937 | 0.900 | 0.928 | 0.967 | 0.935 |
| **PreHD-A** | | | | | | |
| **SPM 8 Unified Segment** | 1.000 |  |  |  |  |  |
| **SPM 8 New Segment** | 0.845 | 1.000 |  |  |  |  |
| **SPM 12 Segment** | 0.908 | 0.764 | 1.000 |  |  |  |
| **ANTs Atropos** | 0.854 | 0.884 | 0.847 | 1.000 |  |  |
| **MALP-EM** | 0.812 | 0.959 | 0.741 | 0.871 | 1.000 |  |
| **FSL FAST** | 0.869 | 0.892 | 0.845 | 0.964 | 0.862 | 1.000 |
| **FreeSurfer** | 0.878 | 0.944 | 0.830 | 0.944 | 0.928 | 0.971 |
| **PreHD-B** | | | | | | |
| **SPM 8 Unified Segment** | 1.000 |  |  |  |  |  |
| **SPM 8 New Segment** | 0.731 | 1.000 |  |  |  |  |
| **SPM 12 Segment** | 0.920 | 0.863 | 1.000 |  |  |  |
| **ANTs Atropos** | 0.785 | 0.941 | 0.931 | 1.000 |  |  |
| **MALP-EM** | 0.856 | 0.956 | 0.940 | 0.943 | 1.000 |  |
| **FSL FAST** | 0.866 | 0.863 | 0.952 | 0.899 | 0.914 | 1.000 |
| **FreeSurfer** | 0.800 | 0.926 | 0.922 | 0.931 | 0.947 | 0.961 |
| **HD1** | | | | | | |
| **SPM 8 Unified Segment** | 1.000 |  |  |  |  |  |
| **SPM 8 New Segment** | 0.762 | 1.000 |  |  |  |  |
| **SPM 12 Segment** | 0.899 | 0.705 | 1.000 |  |  |  |
| **ANTs Atropos** | 0.901 | 0.916 | 0.863 | 1.000 |  |  |
| **MALP-EM** | 0.787 | 0.968 | 0.761 | 0.944 | 1.000 |  |
| **FSL FAST** | 0.908 | 0.853 | 0.881 | 0.937 | 0.881 | 1.000 |
| **FreeSurfer** | 0.904 | 0.884 | 0.902 | 0.949 | 0.925 | 0.970 |
| **HD2** | | | | | | |
| **SPM 8 Unified Segment** | 1.000 |  |  |  |  |  |
| **SPM 8 New Segment** | 0.587 | 1.000 |  |  |  |  |
| **SPM 12 Segment** | 0.836 | 0.768 | 1.000 |  |  |  |
| **ANTs Atropos** | 0.681 | 0.928 | 0.815 | 1.000 |  |  |
| **MALP-EM** | 0.632 | 0.959 | 0.777 | 0.934 | 1.000 |  |
| **FSL FAST** | 0.744 | 0.947 | 0.899 | 0.925 | 0.938 | 1.000 |
| **FreeSurfer** | 0.692 | 0.869 | 0.883 | 0.896 | 0.869 | 0.938 |

*Table 9: Spearman’s ranked correlation for segmentations of occipital GM for all HD participants included in the current study.*

|  | **SPM 8 Unified Segment** | **SPM 8 New Segment** | **SPM 12 Segment** | **ANTs Atropos** | **MALP-EM** | **FSL FAST** |
| --- | --- | --- | --- | --- | --- | --- |
| **Controls** | | | | | | |
| **SPM 8 Unified Segment** | 1.000 |  |  |  |  |  |
| **SPM 8 New Segment** | 0.712 | 1.000 |  |  |  |  |
| **SPM 12 Segment** | 0.907 | 0.854 | 1.000 |  |  |  |
| **ANTs Atropos** | 0.761 | 0.930 | 0.914 | 1.000 |  |  |
| **MALP-EM** | 0.826 | 0.956 | 0.898 | 0.928 | 1.000 |  |
| **FSL FAST** | 0.877 | 0.879 | 0.921 | 0.911 | 0.942 | 1.000 |
| **FreeSurfer** | 0.863 | 0.895 | 0.875 | 0.883 | 0.939 | 0.914 |
| **PreHD-A** | | | | | | |
| **SPM 8 Unified Segment** | 1.000 |  |  |  |  |  |
| **SPM 8 New Segment** | 0.871 | 1.000 |  |  |  |  |
| **SPM 12 Segment** | 0.886 | 0.770 | 1.000 |  |  |  |
| **ANTs Atropos** | 0.893 | 0.859 | 0.793 | 1.000 |  |  |
| **MALP-EM** | 0.859 | 0.940 | 0.794 | 0.887 | 1.000 |  |
| **FSL FAST** | 0.908 | 0.856 | 0.793 | 0.958 | 0.902 | 1.000 |
| **FreeSurfer** | 0.871 | 0.908 | 0.794 | 0.883 | 0.946 | 0.910 |
| **PreHD-B** | | | | | | |
| **SPM 8 Unified Segment** | 1.000 |  |  |  |  |  |
| **SPM 8 New Segment** | 0.553 | 1.000 |  |  |  |  |
| **SPM 12 Segment** | 0.848 | 0.814 | 1.000 |  |  |  |
| **ANTs Atropos** | 0.639 | 0.899 | 0.850 | 1.000 |  |  |
| **MALP-EM** | 0.753 | 0.902 | 0.911 | 0.857 | 1.000 |  |
| **FSL FAST** | 0.815 | 0.737 | 0.902 | 0.812 | 0.848 | 1.000 |
| **FreeSurfer** | 0.657 | 0.774 | 0.868 | 0.762 | 0.862 | 0.764 |
| **HD1** | | | | | | |
| **SPM 8 Unified Segment** | 1.000 |  |  |  |  |  |
| **SPM 8 New Segment** | 0.746 | 1.000 |  |  |  |  |
| **SPM 12 Segment** | 0.958 | 0.741 | 1.000 |  |  |  |
| **ANTs Atropos** | 0.899 | 0.851 | 0.917 | 1.000 |  |  |
| **MALP-EM** | 0.844 | 0.956 | 0.844 | 0.905 | 1.000 |  |
| **FSL FAST** | 0.976 | 0.788 | 0.967 | 0.935 | 0.881 | 1.000 |
| **FreeSurfer** | 0.922 | 0.787 | 0.958 | 0.922 | 0.878 | 0.949 |
| **HD2** | | | | | | |
| **SPM 8 Unified Segment** | 1.000 |  |  |  |  |  |
| **SPM 8 New Segment** | 0.665 | 1.000 |  |  |  |  |
| **SPM 12 Segment** | 0.931 | 0.794 | 1.000 |  |  |  |
| **ANTs Atropos** | 0.755 | 0.892 | 0.841 | 1.000 |  |  |
| **MALP-EM** | 0.785 | 0.959 | 0.877 | 0.850 | 1.000 |  |
| **FSL FAST** | 0.896 | 0.865 | 0.932 | 0.919 | 0.899 | 1.000 |
| **FreeSurfer** | 0.809 | 0.832 | 0.890 | 0.800 | 0.898 | 0.896 |

*Table 10: Spearman’s ranked correlation for segmentations of insula GM for all HD participants included in the current study.*

|  | **SPM 8 Unified Segment** | **SPM 8 New Segment** | **SPM 12 Segment** | **ANTs Atropos** | **MALP-EM** | **FSL FAST** |
| --- | --- | --- | --- | --- | --- | --- |
| **Controls** | | | | | | |
| **SPM 8 Unified Segment** | 1.000 |  |  |  |  |  |
| **SPM 8 New Segment** | 0.830 | 1.000 |  |  |  |  |
| **SPM 12 Segment** | 0.925 | 0.930 | 1.000 |  |  |  |
| **ANTs Atropos** | 0.818 | 0.961 | 0.874 | 1.000 |  |  |
| **MALP-EM** | 0.704 | 0.791 | 0.709 | 0.811 | 1.000 |  |
| **FSL FAST** | 0.839 | 0.939 | 0.868 | 0.953 | 0.837 | 1.000 |
| **FreeSurfer** | 0.832 | 0.883 | 0.819 | 0.890 | 0.884 | 0.925 |
| **PreHD-A** | | | | | | |
| **SPM 8 Unified Segment** | 1.000 |  |  |  |  |  |
| **SPM 8 New Segment** | 0.922 | 1.000 |  |  |  |  |
| **SPM 12 Segment** | 0.928 | 0.853 | 1.000 |  |  |  |
| **ANTs Atropos** | 0.950 | 0.965 | 0.902 | 1.000 |  |  |
| **MALP-EM** | 0.866 | 0.944 | 0.731 | 0.920 | 1.000 |  |
| **FSL FAST** | 0.937 | 0.914 | 0.893 | 0.971 | 0.863 | 1.000 |
| **FreeSurfer** | 0.919 | 0.947 | 0.848 | 0.965 | 0.907 | 0.968 |
| **PreHD-B** | | | | | | |
| **SPM 8 Unified Segment** | 1.000 |  |  |  |  |  |
| **SPM 8 New Segment** | 0.922 | 1.000 |  |  |  |  |
| **SPM 12 Segment** | 0.928 | 0.853 | 1.000 |  |  |  |
| **ANTs Atropos** | 0.950 | 0.965 | 0.902 | 1.000 |  |  |
| **MALP-EM** | 0.866 | 0.944 | 0.731 | 0.920 | 1.000 |  |
| **FSL FAST** | 0.937 | 0.914 | 0.893 | 0.971 | 0.863 | 1.000 |
| **FreeSurfer** | 0.919 | 0.947 | 0.848 | 0.965 | 0.907 | 0.968 |
| **HD1** | | | | | | |
| **SPM 8 Unified Segment** | 1.000 |  |  |  |  |  |
| **SPM 8 New Segment** | 0.878 | 1.000 |  |  |  |  |
| **SPM 12 Segment** | 0.916 | 0.937 | 1.000 |  |  |  |
| **ANTs Atropos** | 0.908 | 0.965 | 0.973 | 1.000 |  |  |
| **MALP-EM** | 0.874 | 0.881 | 0.776 | 0.815 | 1.000 |  |
| **FSL FAST** | 0.938 | 0.958 | 0.979 | 0.982 | 0.851 | 1.000 |
| **FreeSurfer** | 0.887 | 0.929 | 0.928 | 0.928 | 0.866 | 0.959 |
| **HD2** | | | | | | |
| **SPM 8 Unified Segment** | 1.000 |  |  |  |  |  |
| **SPM 8 New Segment** | 0.746 | 1.000 |  |  |  |  |
| **SPM 12 Segment** | 0.829 | 0.790 | 1.000 |  |  |  |
| **ANTs Atropos** | 0.826 | 0.850 | 0.917 | 1.000 |  |  |
| **MALP-EM** | 0.753 | 0.872 | 0.777 | 0.808 | 1.000 |  |
| **FSL FAST** | 0.928 | 0.857 | 0.878 | 0.904 | 0.851 | 1.000 |
| **FreeSurfer** | 0.811 | 0.979 | 0.818 | 0.869 | 0.869 | 0.908 |

*Table 11: Mean change (2011 volume as a percentage of baseline volume), standard deviation and ranges for all tools and groups in frontal lobe GM. Negative values represent increases over time. Results of regression analyses comparing rate of change in controls to HD groups, with significantly greater change in HD groups represented by * p<.05 (light green), **p<.01 (dark green). Age, gender and site were controlled for.*

|  |  | **Controls** | **PreHD-A** | **PreHD-B** | **HD1** | **HD2** |
| --- | --- | --- | --- | --- | --- | --- |
| **SPM 8 Unified Segment** | **% Decrease 2008 to 2011** | 1.24  (3.38) -5.62-8.99 | 2.01  (2.51)  -3.26-6.17 | 1.82  (3.62)  -8.46-8.73 | 4.21  (4.37)  -8.55-11.33 | 4.05  (2.29)  -1.57-7.31 |
|  | **Significant difference** |  | 0.30  (-1.53-2.12)  p = 0.751 | 0.23  (-0.81-1.27)  p = 0.662 | 1.10  (0.34-1.85)  p = 0.004 | 0.38  (-0.12-0.87)  p = 0.137 |
| **SPM 8 New Segment** | **% Decrease 2008 to 2011** | 0.44  (1.50)  -1.75-3.92 | 0.83  (1.13)  -2.16-3.31 | 0.73  (1.03)  -0.85-2.82 | 0.75  (0.90)  -0.94-2.41 | 1.90  (3.11)  -0.32-14.45 |
|  | **Significant difference** |  | 0.32  (-0.35-0.98)  p = 0.349 | 0.12  (-0.22-0.46)  p = 0.488 | 0.18  (-0.04-0.40)  p = 0.115 | 0.23  (-0.16-0.61)  p = 0.250 |
| **SPM 12 Segment** | **% Decrease 2008 to 2011** | 1.15  (2.51)  -5.25-4.17 | 1.69  (4.34)  -9.59-8.19 | 3.22  (2.03)  0.26-6.40 | 4.72  (2.59)  -0.16-9.58 | 5.12  (2.64)  0.87-11.27 |
|  | **Significant difference** | - | 0.22  (-1.84-2.27)  p = 0.838 | 1.03  (0.37-1.69)  p = 0.002 | 1.23  (0.73-1.73)  p = 0.000 | 0.73  (0.29-1.17)  p = 0.001 |
| **ANTs Atropos** | **% Decrease 2008 to 2011** | 2.23  (5.15)  -2.41-22.35 | 2.45  (2.41)  -1.78-8.09 | 1.83  (2.26)  -2.78-5.97 | 3.00  (1.95)  -0.86-6.03 | 3.46  (3.86)  -0.66-17.44 |
|  | **Significant difference** | - | 0.39  (-2.07-2.85)  p = 0.756 | -0.15  (-1.32-1.02)  p = 0.799 | 0.32  (-0.46-1.10)  p = 0.423 | 0.56  (-0.25-1.37)  p = 0.173 |
| **MALP-EM** | **% Decrease 2008 to 2011** | 0.91  (1.98)  -4.01-3.70 | 1.81  (1.38)  -0.36-4.33 | 1.67  (1.71)  -1.03-4.92 | 2.56  (3.01)  -3.95-12.09 | 2.57  (1.91)  0.40-8.28 |
|  | **Significant difference** | - | 0.76  (-0.25-1.77)  p = 0.141 | 0.28  (-0.24-0.79)  p = 0.293 | 0.54  (0.08-1.01)  p = 0.022 | 0.23  (-0.11-0.56)  p = 0.181 |
| **FSL FAST** | **% Decrease 2008 to 2011** | 0.71  (3.59)  -5.53-8.23 | 3.62  (5.60)  -6.95-18.21 | 2.52  (4.06)  -4.16-13.94 | 3.60  (5.91)  -7.84-12.75 | 7.74  (9.14)  -3.01-32.70 |
|  | **Significant difference** | - | 1.70  (0.00-3.39)  p = 0.050 | 0.94  (0.19-1.68)  p = 0.014 | 0.77  (0.20-1.34)  p = 0.008 | 0.96  (0.21-1.71)  p = 0.012 |
| **FreeSurfer** | **% Decrease 2008 to 2011** | 0.55  (1.68)  -1.68-3.49 | 2.11  (1.84)  -1.37-6.96 | 2.05  (2.28)  -1.59-6.33 | 3.14  (2.43)  -1.39-7.06 | 3.41  (2.00)  0.26-6.45 |
|  | **Significant difference** |  | 1.35  (0.30-2.41)  p = 0.012 | .71  (0.17-1.24)  p=.010 | .86  (.48-1.23)  p = .000 | .61  (.31-.91)  p = .000 |

*Table 12: Mean change (2011 volume as a percentage of baseline volume), standard deviation and ranges for all tools and groups in temporal lobe GM. Negative values represent increases over time. Results of regression analyses comparing rate of change in controls to HD groups, with significantly greater change in HD groups represented by * p<.05 (light green), **p<.01 (dark green). Age, gender and site were controlled for.*

|  |  | **Controls** | **PreHD-A** | **PreHD-B** | **HD1** | **HD2** |
| --- | --- | --- | --- | --- | --- | --- |
| SPM 8 Unified Segment | **% Decrease 2008 to 2011** | 1.47  (3.00)  -5.72-7.56 | 1.92  (1.93)  -1.79-4.96 | 1.27  (4.02)  -10.16-7.24 | 4.27  (3.82)  -6.00-11.03 | 3.82  (2.42)  -0.65-6.72 |
|  | **Significant difference** | - | 0.46  (-1.12-2.03)  p = 0.569 | -0.07  (-1.06-0.91)  p = 0.887 | 0.90  (0.25-1.55)  p = 0.006 | 0.36  (-0.10-0.83)  p = 0.122 |
| SPM 8 New Segment | **% Decrease 2008 to 2011** | 0.75  (1.53)  -1.63-3.97 | 0.44  (1.29)  -1.67-3.40 | 0.39  (0.99)  -2.22-1.71 | 0.70  (1.21)  -1.97-4.11 | 1.48  (3.90)  -3.90-16.23 |
|  | **Significant difference** | - | -0.16  (-0.95-0.63)  p = 0.692 | -0.16  (-0.48-0.16)  p = 0.330 | -0.06  (-0.31-0.18)  p = 0.617 | 0.05  (-0.40-0.49)  p = 0.839 |
| SPM 12 Segment | **% Decrease 2008 to 2011** | 1.41  (2.35)  -5.56-5.02 | 1.49  (3.21)  -5.70-6.56 | 2.49  (2.03)  -1.48-6.04 | 4.31  (2.58)  0.70-10.08 | 4.80  (3.20)  -0.51-10.75 |
|  | **Significant difference** | - | -0.13  (-1.81-1.55)  p = 0.882 | 0.56  (-0.04-1.16)  p = 0.067 | 0.88  (0.43-1.32)  p = 0.000 | 0.60  (0.16-1.03)  p = 0.008 |
| ANTs Atropos | **% Decrease 2008 to 2011** | 2.63  (3.50)  -0.91-15.76 | 2.11  (2.80)  -2.32-8.83 | 1.64  (1.98)  -1.96-5.58 | 3.06  (1.81)  -0.02-6.48 | 3.43  (4.62)  -2.50-19.77 |
|  | **Significant difference** | - | -0.11  (-1.95-1.72)  p = 0.904 | -0.42  (-1.18-0.35)  p = 0.284 | 0.10  (-0.44-0.64)  p = 0.721 | 0.47  (-0.18-1.12)  p = 0.160 |
| MALP-EM | **% Decrease 2008 to 2011** | 1.26  (1.54)  -3.59-4.26 | 1.29  (1.75)  -1.64-5.62 | 1.28  (1.54)  -1.49-5.11 | 2.34  (3.95)  -8.26-14.21 | 2.19  (3.20)  -4.65-12.75 |
|  | **Significant difference** | - | -0.03  (-1.02-0.95)  p = 0.950 | 0.00  (-0.40-0.40)  p = 0.998 | 0.19  (-0.38-0.77)  p = 0.512 | 0.04  (-0.35-0.43)  p = 0.829 |
| FSL FAST | **% Decrease 2008 to 2011** | 1.47  (3.32)  -5.72-7.87 | 3.72  (4.27)  -3.02-10.72 | 2.13  (2.79)  -1.40-11.11 | 4.38  (4.92)  -3.65-17.89 | 5.13  (3.90)  -1.31-14.52 |
|  | **Significant difference** | - | 1.33  (-0.41-3.07)  p = 0.135 | 0.43  (-0.30-1.15)  p = 0.248 | 0.65  (0.05-1.25)  p = 0.035 | 0.34  (-0.09-0.78)  p = 0.124 |
| FreeSurfer | **% Decrease 2008 to 2011** | 1.18 (2.43) -3.25-7.54 | 1.49  (2.55)  -4.84-6.39 | 1.58  (1.92)  -1.87-5.11 | 3.42  (1.73)  1.19-7.38 | 4.04  (2.45)  -2.29-7.79 |
|  | **Significant difference** |  | .23  (-1.26-1.72)  p = 0.762 | .28  (-.32-.87)  p = 0.364 | .71  (.71-1.12)  p = 0.001 | .63  (.24-1.02)  p = 0.002 |

*Table 13: Mean change (2011 volume as a percentage of baseline volume), standard deviation and ranges for all tools and groups in parietal lobe GM. Negative values represent increases over time. Results of regression analyses comparing rate of change in controls to HD groups, with significantly greater change in HD groups represented by * p<.05 (light green), **p<.01 (dark green). Age, gender and site were controlled for.*

|  |  | **Controls** | **PreHD-A** | **PreHD-B** | **HD1** | **HD2** |
| --- | --- | --- | --- | --- | --- | --- |
| SPM 8 Unified Segment | **% Decrease 2008 to 2011** | 0.44  (3.19)  -4.34-8.36 | 2.24  (2.06)  -2.31-5.63 | 2.13  (3.59)  -6.23-9.01 | 4.60  (4.06)  -7.72-10.10 | 3.81  (2.69)  -1.66-7.53 |
|  | **Significant difference** | - | 1.62  (-0.06-3.31)  p = 0.059 | 0.85  (-0.21-1.90)  p = 0.115 | 1.48 (  0.76-2.19)  p = 0.000 | 1.62  (-0.06-3.31)  p = 0.059 |
| SPM 8 New Segment | **% Decrease 2008 to 2011** | -0.37  (1.67)  -3.77-2.14 | 0.81  (0.97)  -1.07-2.37 | 0.60  (0.84)  -1.24-1.86 | 1.05  (1.13)  -1.31-2.93 | 1.56  (2.29)  0.03-10.58 |
|  | **Significant difference** | - | 1.05  (0.36-1.74)  p = 0.003 | 0.45  (0.11-0.78)  p = 0.009 | 0.55  (0.31-0.80)  p = 0.000 | 0.36  (0.04-0.67)  p = 0.027 |
| SPM 12 Segment | **% Decrease 2008 to 2011** | 0.51  (3.00)  -7.75-4.38 | 1.70  (3.61)  -8.07-7.88 | 3.33  (1.48)  0.91-6.51 | 5.22  (2.28)  1.25-9.39 | 4.95  (2.91)  0.59-9.92 |
|  | **Significant difference** | - | 0.83  (-1.15-2.81)  p = 0.412 | 1.42  (0.75-2.10)  p = 0.000 | 1.61  (1.06-2.15)  p = 0.000 | 0.80  (0.30-1.30)  p = 0.002 |
| ANTs Atropos | **% Decrease 2008 to 2011** | 0.47  (1.94)  -2.63-5.38 | 1.98  (2.68)  -4.78-7.28 | 2.46  (2.28)  -1.66-8.58 | 3.54  (2.10)  -0.37-6.95 | 3.30  (3.24)  -2.03-12.84 |
|  | **Significant difference** | - | 1.29  (-0.18-2.75)  p = 0.085 | 0.96  (0.35-1.57)  p = 0.002 | 1.11  (0.74-1.48)  p = 0.000 | 0.68  (0.22-1.13)  p = 0.003 |
| MALP-EM | **% Decrease 2008 to 2011** | 0.07  (1.57)  -3.95-2.40 | 0.97  (1.00)  -1.03-3.07 | 1.39  (1.22)  -0.65-3.56 | 2.23  (1.68)  -0.59-7.16 | 1.94  (1.55)  -0.60-6.01 |
|  | **Significant difference** | - | 0.77  (0.05-1.48)  p = 0.036 | 0.60  (0.21-0.99)  p = 0.003 | 0.76  (0.48-1.03)  p = 0.000 | 0.34  (0.08-0.60)  p = 0.011 |
| FSL FAST | **% Decrease 2008 to 2011** | 0.09  (3.41)  -9.04-5.46 | 3.27  (3.93)  -2.64-11.00 | 3.15  (2.96)  -0.48-11.11 | 4.24  (3.65)  -4.03-11.29 | 5.30  (4.42)  0.72-17.44 |
|  | **Significant difference** | - | 2.23  (0.54-3.92)  p = 0.010 | 1.54  (0.77-2.32)  p = 0.000 | 1.25  (0.72-1.78)  p = 0.000 | 0.80  (0.24-1.36)  p = 0.005 |
| FreeSurfer | **% Decrease 2008 to 2011** | 0.45  (1.41)  -2.55-2.50 | 1.45  (1.94)  -2.51-5.97 | 2.96  (2.14)  -1.60-7.27 | 3.89  (2.54)  0.33-9.60 | 3.86  (2.38)  -0.78-9.35 |
|  | **Significant difference** |  | .60  (-.40-1.61)  p = 0.239 | 1.21  (.70-1.72)  p = 0.000 | 1.06  (.67-1.44)  p = 0.000 | .65  (0.35-0.94)  p = 0.000 |

*Table 14: Mean change (2011 volume as a percentage of baseline volume), standard deviation and ranges for all tools and groups in occipital lobe GM. Negative values represent increases over time. Results of regression analyses comparing rate of change in controls to HD groups, with significantly greater change in HD groups represented by * p<.05 (light green), **p<.01 (dark green). Age, gender and site were controlled for.*

|  |  | **Controls** | **PreHD-A** | **PreHD-B** | **HD1** | **HD2** |
| --- | --- | --- | --- | --- | --- | --- |
| SPM 8 Unified Segment | **% Decrease 2008 to 2011** | 0.35  (4.42)  -11.14-9.57 | 2.18  (3.12)  -4.03-8.78 | 1.88  (4.26)  -8.03-9.34 | 6.06  (4.87)  -7.32-15.50 | 5.01  (3.26)  -0.43-9.77 |
|  | **Significant difference** | - | 1.81  (-0.79-4.41)  p = 0.173 | 0.90  (-0.46-2.26)  p = 0.197 | 1.95  (1.01-2.89)  p = 0.000 | 1.31  (0.48-2.14)  p = 0.002 |
| SPM 8 New Segment | **% Decrease 2008 to 2011** | -0.66  (2.13)  -7.72-1.27 | 0.96  (1.95)  -3.02-4.27 | 0.39  (1.60)  -1.81-3.56 | 1.78  (1.22)  -0.16-4.12 | 2.40  (4.68)  -2.66-20.47 |
|  | **Significant difference** | - | 1.85  (0.60-3.11)  p = 0.004 | 0.56  (0.01-1.11)  p = 0.047 | 0.83  (0.47-1.19)  p = 0.000 | 0.76  (0.18-1.34)  p = 0.010 |
| SPM 12 Segment | **% Decrease 2008 to 2011** | 0.58  (3.47)  -9.26-4.94 | 2.28  (5.04)  -7.40-11.37 | 3.88  (2.72)  -0.14-9.25 | 7.11  (3.24)  2.44-15.85 | 7.12  (3.32)  1.68-12.39 |
|  | **Significant difference** | - | 1.59  (-1.11-4.30)  p = 0.248 | 1.74  (0.83-2.66)  p = 0.000 | 2.15  (1.47-2.83)  p = 0.000 | 1.42  (0.82-2.03)  p = 0.000 |
| ANTs Atropos | **% Decrease 2008 to 2011** | 0.35  (2.95)  -8.51-6.80 | 1.90  (3.98)  -3.01-8.94 | 2.79  (2.95)  -2.22-8.53 | 4.64  (2.58)  0.59-10.90 | 4.65  (5.08)  -2.34-22.64 |
|  | **Significant difference** | - | 1.69  (-0.44-3.82)  p = 0.119 | 1.32  (0.46-2.18)  p = 0.003 | 1.45  (0.91-1.98)  p = 0.000 | 1.21  (0.51-1.91)  p = 0.001 |
| MALP-EM | **% Decrease 2008 to 2011** | -0.73  (2.10)  -7.74-1.90 | 1.10  (2.05)  -3.06-4.50 | 1.52  (2.16)  -4.24-5.23 | 3.57  (2.56)  -0.53-9.38 | 2.99  (3.04)  -3.59-11.72 |
|  | **Significant difference** | - | 1.93  (0.66-3.19)  p = 0.003 | 1.17  (0.54-1.80)  p = 0.000 | 1.36  (0.90-1.81)  p = 0.000 | 0.92  (0.48-1.36)  p = 0.000 |
| FSL FAST | **% Decrease 2008 to 2011** | 0.63  (6.00)  -18.17-13.82 | 5.17  (6.56)  -3.67-22.14 | 4.22  (6.99)  -0.25-31.20 | 9.45  (10.13)  0.13-44.52 | 12.09  (12.81)  -1.89-37.55 |
|  | **Significant difference** | - | 3.47  (0.52-6.41)  p = 0.021 | 2.13  (0.71-3.55)  p = 0.003 | 2.26  (0.99-3.53)  p = 0.000 | 1.82  (0.75-2.88)  p = 0.001 |
| FreeSurfer | **% Decrease 2008 to 2011** | -0.08  (2.84)  -10.87-2.81 | 1.68  (2.54)  -3.08-6.66 | 3.06  (2.99)  -3.23-7.75 | 4.71  (2.49)  -1.62-8.27 | 4.96  (3.43)  0.17-11.70 |
|  | **Significant difference** |  | 1.74  (0.12-3.37)  p = 0.036 | 1.71  (0.88-2.54)  p = 0.000 | 1.57  (1.02-2.11)  p = 0.000 | 1.28  (0.73-1.84)  p = 0.000 |

*Table 15: Mean change (2011 volume as a percentage of baseline volume), standard deviation and ranges for all tools and groups in insula GM. Negative values represent increases over time. Results of regression analyses comparing rate of change in controls to HD groups, with significantly greater change in HD groups represented by * p<.05 (light green), **p<.01 (dark green). Age, gender and site were controlled for.*

|  |  | **Controls** | **PreHD-A** | **PreHD-B** | **HD1** | **HD2** |
| --- | --- | --- | --- | --- | --- | --- |
| SPM 8 Unified | **% Decrease 2008 to 2011** | 0.28  (3.39)  -9.01-5.73 | 1.50  (2.35)  –3.92-5.17 | 1.68  (2.59)  -3.62-5.56 | 3.97  (3.19)  -4.54-8.96 | 4.11  (3.36)  -2.20-13.00 |
|  | **Significant difference** | - | 0.75  (-1.14-2.65)  p = 0.438 | 0.74  (-0.19-1.67)  p = 0.118 | 1.22  (0.58-1.86)  p = 0.000 | 1.07  (0.44-1.70)  p = 0.001 |
| SPM 8 New | **% Decrease 2008 to 2011** | -0.77  (2.95)  -11.79-2.19 | 0.17  (1.20)  -2.01-1.94 | 0.54  (1.69)  -2.97-4.18 | 0.23  (1.24)  -3.30-2.12 | 1.35  (5.08)  -3.15-21.44 |
|  | **Significant difference** | - | 0.86  (-0.51-2.23)  p = 0.218 | 0.65  (-0.05-1.35)  p = 0.070 | 0.32  (-0.12-0.77)  p = 0.154 | 0.62  (-0.09-1.33)  p = 0.086 |
| SPM 12 | **% Decrease 2008 to 2011** | -0.13  (3.01)  -9.85-3.30 | 1.55  (3.02)  -5.67-7.31 | 2.64  (1.84)  0.00-6.52 | 4.49  (2.42)  -0.47-8.85 | 5.27  (4.37)  0.22-16.80 |
|  | **Significant difference** | - | 1.39  (-0.45-3.24)  p = 0.138 | 1.41  (0.68-2.14)  p = 0.000 | 1.52  (0.98-2.06)  p = 0.000 | 1.32  (0.65-1.99)  p = 0.000 |
| Atropos | **% Decrease 2008 to 2011** | -0.23  (3.71)  -13.19-4.79 | 0.25  (2.10)  -4.82-3.59 | 0.51  (1.66)  -3.63-3.92 | 1.85  (1.73)  -0.97-4.60 | 3.16  (5.20)  -1.54-21.45 |
|  | **Significant difference** | - | 0.41  (-1.33-2.14)  p = 0.645 | 0.40  (-0.44-1.23)  p = 0.351 | 0.69  (0.18-1.20)  p = 0.008 | 1.20  (0.42-1.98)  p = 0.003 |
| MALPEM | **% Decrease 2008 to 2011** | -1.65  (6.72)  -23.50-10.04 | 1.07  (2.94)  -1.91-10.41 | 1.18  (3.22)  -2.53-12.26 | 1.07  (4.21)  -8.27-8.34 | 1.47  (3.70)  -3.90-11.62 |
|  | **Significant difference** | - | 2.96  (-0.16-6.08)  p = 0.063 | 1.30  (-0.27-2.86)  p = 0.104 | 0.72  (-0.37-1.82)  p = 0.196 | 0.77  (-0.24-1.78)  p = 0.136 |
| FAST | **% Decrease 2008 to 2011** | -0.72  (5.10)  -19.77-4.00 | 1.99  (2.83)  -2.61-8.31 | 1.71  (2.12)  -1.16-8.53 | 2.90  (1.91)  -1.34-5.73 | 3.49  (2.41)  0.59-9.00 |
|  | **Significant difference** | - | 1.97  (-0.11-4.04)  p = 0.063 | 1.19  (0.13-2.25)  p = 0.028 | 1.13  (0.44-1.81)  p = 0.001 | 0.93  (0.21-1.64)  p = 0.011 |
| FreeSurfer | **% Decrease 2008 to 2011** | -0.69  (4.26)  -16.16-3.23 | 1.29  (2.49)  -1.88-7.00 | 1.13  (2.47)  -4.12-5.36 | 1.74  (2.42)  -1.79-6.90 | 3.43  (3.51)  -3.24-14.01 |
|  | **Significant difference** |  | 1.56  (-0.45-3.56)  p = 0.128 | 0.84  (-0.18-1.87)  p = 0.106 | 0.72  (0.03-1.41)  p = 0.041 | 0.95  (0.27-1.63)  p = 0.006 |
